# Effects of Coarse Graining and Saturation of Hydrocarbon Chains on Structure and Dynamics of Simulated Lipid Molecules

**DOI:** 10.1038/s41598-017-11761-5

**Published:** 2017-09-13

**Authors:** Pavel Buslaev, Ivan Gushchin

**Affiliations:** 1 Moscow Institute of Physics and Technology, 141700 Dolgoprudny, Russia; 2 Institute of Complex Systems (ICS), ICS-6: Structural Biochemistry, Research Centre Jülich, 52425 Jülich, Germany

## Abstract

Molecular dynamics simulations are used extensively to study the processes on biological membranes. The simulations can be conducted at different levels of resolution: all atom (AA), where all atomistic details are provided; united atom (UA), where hydrogen atoms are treated inseparably of corresponding heavy atoms; and coarse grained (CG), where atoms are grouped into larger particles. Here, we study the behavior of model bilayers consisting of saturated and unsaturated lipids DOPC, SOPC, OSPC and DSPC in simulations performed using all atom CHARMM36 and coarse grained Martini force fields. Using principal components analysis, we show that the structural and dynamical properties of the lipids are similar, both in AA and CG simulations, although the unsaturated molecules are more dynamic and favor more extended conformations. We find that CG simulations capture 75 to 100% of the major collective motions, overestimate short range ordering, result in more flexible molecules and 5–7 fold faster sampling. We expect that the results reported here will be useful for comprehensive quantitative comparisons of simulations conducted at different resolution levels and for further development and improvement of CG force fields.

## Introduction

Nowadays, molecular dynamics (MD) simulations became indispensable in biological research. They are used to study the properties of solvents, small molecules, polymers, oligonucleotides and proteins. The simulations are especially fruitful in the field of membrane biology where they can be used to characterize the behavior of various lipidic phases: micelles, bilayers, vesicles, cubic and others, and to model physiological processes, such as membrane pore formation or membrane fusion and fission, and to probe the effects of additives^1–4^.

One of prerequisites of a successful simulation is employment of a correct set of simulation parameters, known as a force field (FF). Currently, several FFs for simulations of lipids are available^5–20^. Since the parameterization is often based on different principles, inevitably there are differences in the results of the simulations, which however become smaller in later versions of popular FFs^21–29^. Among the major differences between the FFs is the resolution level at which the lipids are simulated. First, there are all atom (AA) FFs, such as CHARMM36^5^, Lipid14^6^, OPLS-AA^7, 8^ and Stockholm lipids^9^. These FFs faithfully represent both structure and dynamics of lipid molecules, but require significant computational power for simulations of complex phenomena. Next, it is possible to treat some or all of the hydrogen atoms inseparably from the heavy atoms to which they are covalently bound, and consequently reduce the number of interacting particles and computational cost. This so-called united atom (UA) approach was implemented in, for example, Berger^10^, GROMOS^11–13^, CHARMM36-UA^14^ and other FFs^15, 16^, and results in 1.5-2x speedup of the calculations.

Unfortunately, many relevant physiological processes occur on spatiotemporal scales currently unreachable for atomistic FFs, and sampling is a problem in MD simulations^30^. Therefore, a set of even simpler coarse grained (CG) models has been developed. In such models, molecule’s atoms are grouped into beads, so that the general geometry and connectivity are retained^31–35^. These models were used successfully to simulate proteins^36, 37^ as well as lipids. There are several CG FFs for lipid molecules that illustrate the force matching approach^38–40^, reproduce characteristic time scales from AA simulations and experiments^41, 42^ and concentrate on the phase behavior of lipid membranes^43, 44^ or interactions of proteins with lipids^45^. Perhaps the most popular lipid CG FF is Martini^17–19^, now expanded to proteins^46, 47^, DNA^48^ and carbohydrates^49^. With Martini, it is possible to study extremely large and diverse systems such as plasma or thylakoid membranes^50, 51^, cellulose microfibers^52^, and many others.

CG FFs are very useful for extended simulations, and largely capture the major structural and dynamical features of atomistic FFs^53–56^. Inevitably, there are also some limitations. The short-range ordering is overestimated^53, 54^. The lipid-lipid interactions in Martini are too weak, water-lipid repulsion is overestimated, and the temperature dependencies of thermodynamic quantities are weaker compared to AA FFs^53^. The dynamics is altered: convergence of configurational entropy and full sampling of internal motions of lipid tails in CG simulations can be reached faster than in atomistic simulations^55, 56^. Dynamics of water particles and peptides in Martini simulations was found to be ~4 times faster than that in the AA and UA simulations^19, 57, 58^. Finally, uneven acceleration of dynamic processes was also observed in other CG simulations, and thus the characteristic time scales were deemed unrealistic^59^.

**Figure 1:**
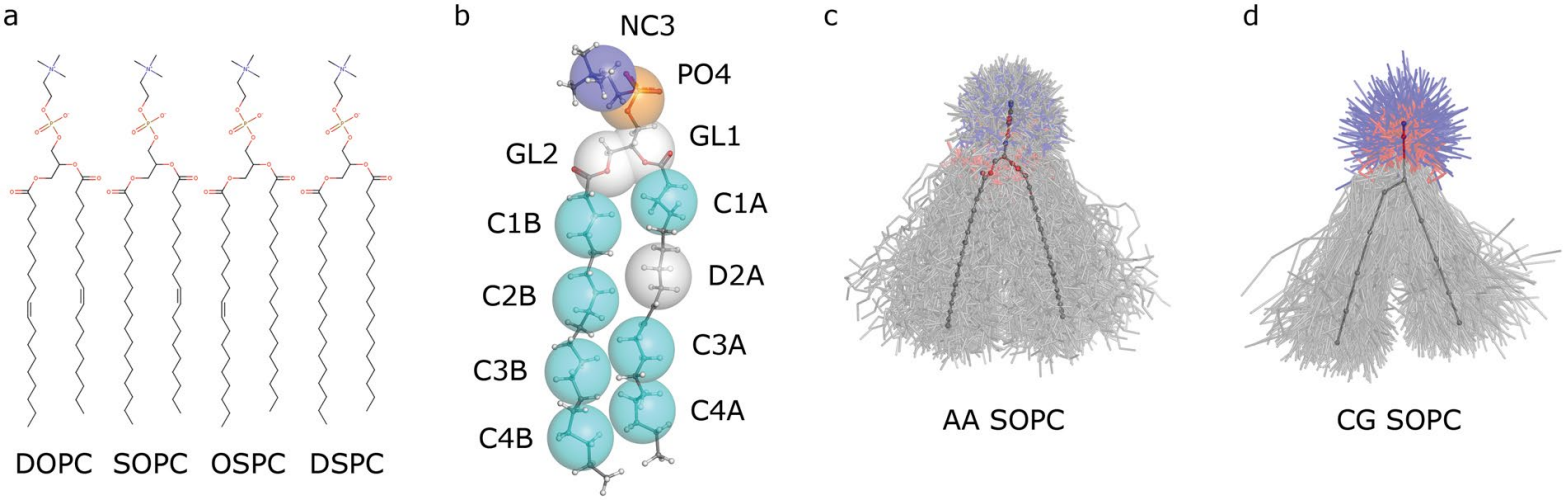
Structures of the studied molecules. (**a**) Chemical structures of DOPC (1,2-dioleoyl-*sn*-glycero-3-phosphocholine, 18:1c9 PC), SOPC (1-stearoyl-2-oleoyl-*sn*-glycero-3-phosphocholine, 18:0/18:1c9 PC), OSPC (1-oleoyl-2-stearoyl-*sn*-glycero-3-phosphocholine, 18:1c9/18:0 PC) and DSPC (1,2-distearoyl-*sn*-glycero-3-phosphocholine, 18:0 PC), respectively. (**b**) Martini mapping between all-atom and coarse grained structures for SOPC lipid molecule. Mappings for DOPC, SOPC, OSPC and DSPC headgroups and oleoyl and stearoyl chains are identical. (**c**,**d**) Samples of SOPC conformations observed in the AA and CG molecular dynamics simulations. 500 structure snapshots from each simulation are shown. The structures are aligned to the averaged structure.

One of the defining factors of lipid molecule properties is the saturation of its hydrocarbon chains. The model lipids with different combinations of saturated and mono-unsaturated hydrocarbon chains are 1,2-dioleoyl-*sn*-glycero-3-phosphocholine (18:1c9 PC, DOPC), 1-stearoyl-2-oleoyl-*sn*-glycero-3-phosphocholine (18:0/18:1c9 PC, SOPC), 1-oleoyl-2-stearoyl-*sn*-glycero-3-phosphocoline (18:1c9/18:0 PC, OSPC) and 1,2-distearoyl-*sn*-glycero-3-phosphocholine (18:0 PC, DSPC) (Fig. 1a). Mono-unsaturated chains with *cis* double bonds have higher propensity to be disordered than the saturated ones, and consequently the more saturated chains such a lipid has, the higher is its melting temperature (256, 279, 282 and 328 K for DOPC, SOPC, OSPC and DSPC, respectively^60^), and the lower is its area per lipid (~73, ~70, ~70 and 67 Å^2^ at for DOPC, SOPC, OSPC and DSPC, respectively, at 338 K^61–70^). These effects can be successfully reproduced in MD simulations of lipid bilayers^63, 64, 71^.

Recently, we applied the principal components analysis (PCA)^72^ to compare several AA and UA lipid forcefields^28^. Here, we demonstrate how the PCA approach can be utilized to study the influence of coarse graining and saturation of hydrocarbon chains on structure and dynamics of lipid molecules in molecular dynamics simulations. We compare the properties of DOPC, SOPC, OSPC and DSPC molecules in atomistic and CG simulations at the single molecule level and describe the similarities and differences between the simulations. Most of the simulations are conducted at 338 K, above the DSPC melting temperature. However, this is an elevated temperature for most of the biological systems, and many of the experiments and simulations are performed at or below 310 K. Consequently, we probe the behavior of DOPC also at 323 K and 310 K, and analyze the influence of the simulation temperature on the lipid’s conformations and dynamics.

## Results and Discussion

### Performed simulations

We performed molecular dynamics simulations of lipids DOPC, SOPC, OSPC, and DSPC that have different combinations of monounsaturated (18:1c9) oleoyl and saturated (18:0) stearoyl chains at positions *sn*-1 and *sn*-2 (Fig. 1a), at two different levels of resolution: AA and CG, using CHARMM36^5^ and Martini^17–19^ force fields, respectively. Details of the simulations are presented in Table 1. Simulations AA1-3 and CG1-3 probe the effects of temperature on dynamics of individual lipid molecules, whereas AA1,4-6 and CG1,4-6 probe the similarities and differences between the studied lipids. Chemical structures, observed conformations and Martini mapping between the AA and CG models are presented in Fig. 1. Since macroscopic properties of DOPC, SOPC, OSPC and DSPC in atomistic and CG simulations have been described elsewhere^5, 9, 18, 65, 68–70^, we focus on the properties of individual molecules.

**Table 1:** Overview of the simulations presented in this study.

Simulation	Lipid	Temperature, K	No. of lipids	No. of waters *	Force field	Water model	Duration, ns
AA1	DOPC	338	128	5120	CHARMM36	CHARMM TIP3P	200
AA2	DOPC	323	128	5120	CHARMM36	CHARMM TIP3P	200
AA3	DOPC	310	128	5120	CHARMM36	CHARMM TIP3P	200
AA4	SOPC	338	128	5120	CHARMM36	CHARMM TIP3P	200
AA5	OSPC	338	128	5120	CHARMM36	CHARMM TIP3P	200
AA6	DSPC	338	128	5120	CHARMM36	CHARMM TIP3P	200
CG1	DOPC	338	128	1276	Martini	Polarizable Martini	200
CG2	DOPC	323	128	1276	Martini	Polarizable Martini	200
CG3	DOPC	310	128	1276	Martini	Polarizable Martini	200
CG4	SOPC	338	128	1276	Martini	Polarizable Martini	200
CG5	OSPC	338	128	1276	Martini	Polarizable Martini	200
CG6	DSPC	338	128	1276	Martini	Polarizable Martini	200

*For Martini simulations, the number of polarizable water particles. Each Martini water particle corresponds to 4 water molecules.

### Average structures

**Figure 2:**
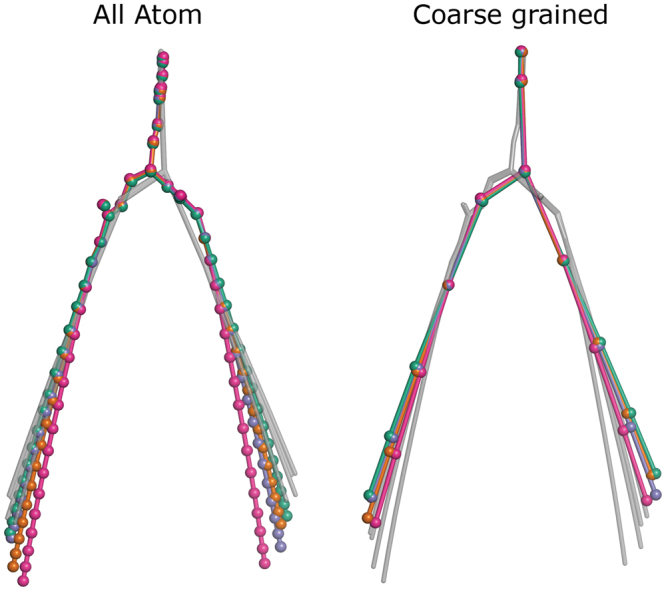
Influence of hydrocarbon chain saturation on the average structure of lipid molecule. DOPC is shown in marine, SOPC is in orange, OSPC is in violet and DSPC is in magenta. Average structures at another level of resolution are shown in grey. While the headgroup structures are well conserved, the positions of hydrophobic chains differ. Oleoyl (18:1c9) chains occupy the outermost position and stearoyl (18:0) chains are the innermost.

First, we calculated the average atomic positions (Fig. 2). Notably, both in AA and CG simulations, the unsaturated acyl chains occupy the outermost position while the saturated acyl chains occupy the innermost one (Fig. 2). This is most probably due to the fact that the unsaturated hydrocarbon chains are less ordered^63^ and deviate from the normal to the bilayer. As for the individual atoms, in the AA simulations, positions of DOPC oleoyl chains are almost identical to the positions of SOPC and OSPC oleoyl chains. Similarly, positions of DSPC stearoyl chains are almost identical to those of SOPC and OSPC stearoyl chains. The average CG structures correspond to the average AA structures, in accordance with the Martini atoms-to-beads mapping, and reveal the same trends.

### Principal components analysis

To gain further insight into the effects of coarse graining and saturation of hydrocarbon chains, we studied the lipid conformations observed in the obtained trajectories using principal components analysis^28, 72^. The analysis results in a linear transformation of the conformational space via identification of new orthogonal basis vectors, called principal components (PCs), such that the variance of the input data along PC1 is the greatest possible, the variance along PC2 is the second greatest, etc. Each of the principal components defines a certain collective motion of the molecule’s atoms.

Similarly to our previous analysis of AA and UA DOPC force fields, we observed that the covariance matrices of atomic displacements are dominated by few large-amplitude motions (Figure S2)^28^. Four major components account for ~50% of the structural variation, and 90% of the structural variation is covered by 14 components for the AA simulations, and by 11 components for the CG simulations. This slightly lower dimensionality of the CG configurational space is not unexpected^55, 56^. As for the dependence on the hydrophobic chain saturation, the eigenvalue distributions are similar for the four lipids, although there is a clear trend that for more saturated hydrophobic chains the contribution of the major principal components becomes higher (Figure S2).

In all of the simulations the first, largest-amplitude principal component (PC1) corresponds to the scissoring motion of the hydrophobic tails (Figure S3). The others, lower-amplitude components, are less collective^28^ and account for various bending and twisting motions of lipid molecules (Figure S3). Whereas in the atomistic simulations the principal components almost always include displacements both in the plane of the scissoring PC1 motion and perpendicular to it, in CG simulations the displacements are almost always either completely in plane or completely perpendicular to it (Figure S3).

### Comparison of the simulations

Direct comparison of the obtained AA and CG trajectories to each other using PCA is problematic since the AA and CG lipid representations have different number of particles. This can be overcome in two ways. First, for most of the analyses we convert the AA trajectories into Martini CG representation. Second, for comparisons of AA collective motions with CG collective motions, we conduct PCA on the AA trajectories, and only then convert the obtained principal components into CG representation.

**Figure 3:**
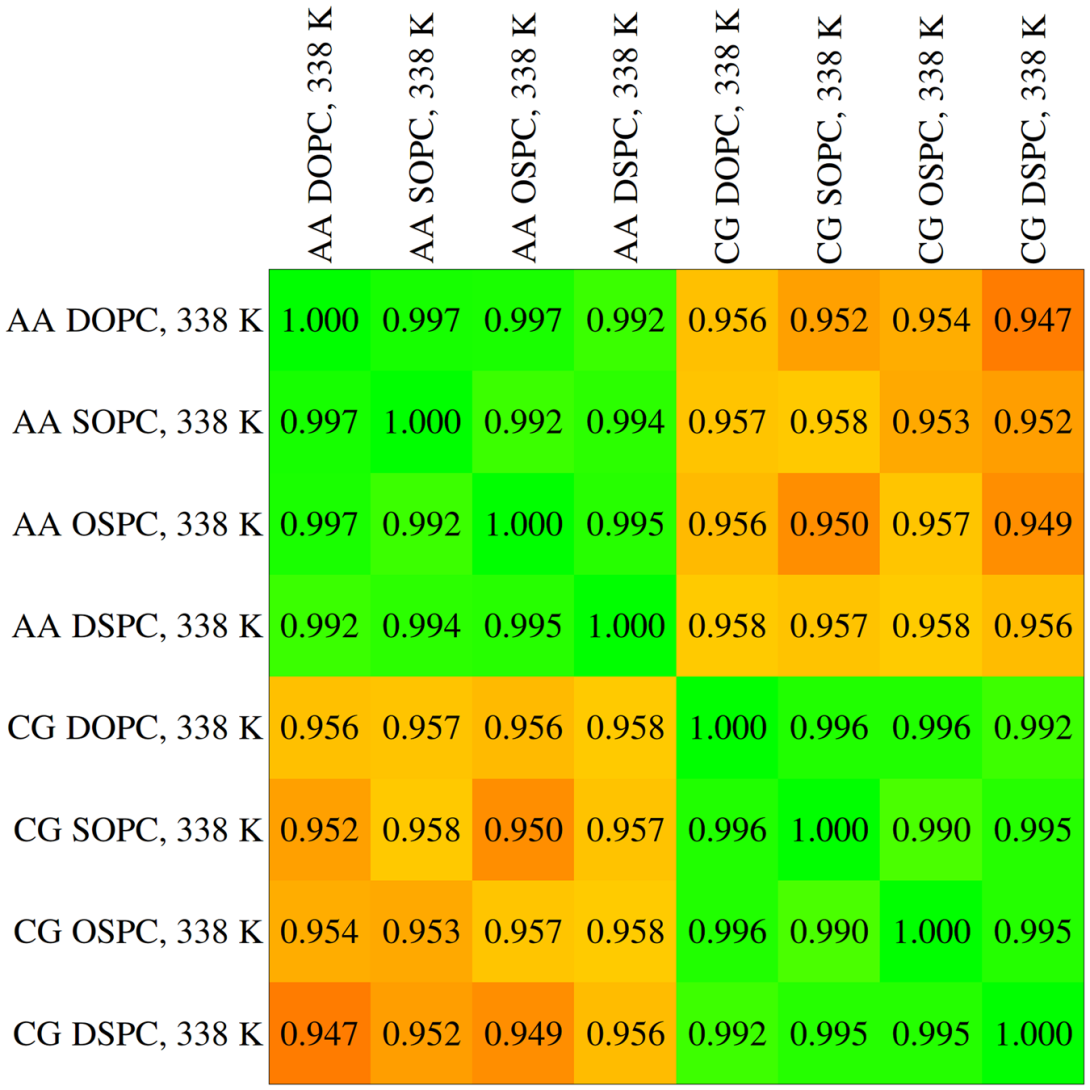
Pearson correlation coefficients between the covariance matrices of atomic displacements in respective simulations^28^ (please find the description in Methods). The correlation coefficients are color-coded to highlight the similarity and dissimilarity between the simulations.

A very general comparison of the conformational ensembles obtained in different simulations can be performed by calculating the Pearson correlation coefficients (PCCs) of the covariance matrix elements^28^. Analysis of the coarse grained trajectories reveals that AA simulations produce very similar covariance values that are however different from those obtained in CG simulations (Fig. 3). When compared in the common AA basis, AA covariance matrices produce PCC values similar to those obtained in CG basis (Figure S4). Comparison of present DOPC simulations at 310 K with those from our previous work^28^ reveals better correspondence between Martini and latest AA force fields (CHARMM36, Lipid14 and Slipids) than between Martini and Berger/GROMOS family UA forcefields to which Martini was originally compared^17, 18^ (Figure S5). Finally, both in AA and CG simulations, DOPC-SOPC differences are the same as DOPC-OSPC, SOPC-DSPC and OSPC-DSPC differences, and DOPC-DSPC differences are the same as SOPC-OSPC differences (Figs 3 and S4). Interestingly, covariance matrices for DOPC simulated at different temperatures (310–338 K) show very little change both in AA and CG simulations (Figure S6). Correspondence between the covariance matrices obtained in AA and CG simulations is slightly better for the CG simulations conducted at 338 K (Figure S6).

To find the differences in behavior of the four lipids, we compared the principal components at each level of resolution. The dot product matrices are almost diagonal, and the DOPC-DSPC and OSPC-SOPC differences are, as expected, larger than those between DOPC and SOPC/OSPC, or between SOPC/OSPC and DSPC (Figure S7).

**Figure 4:**
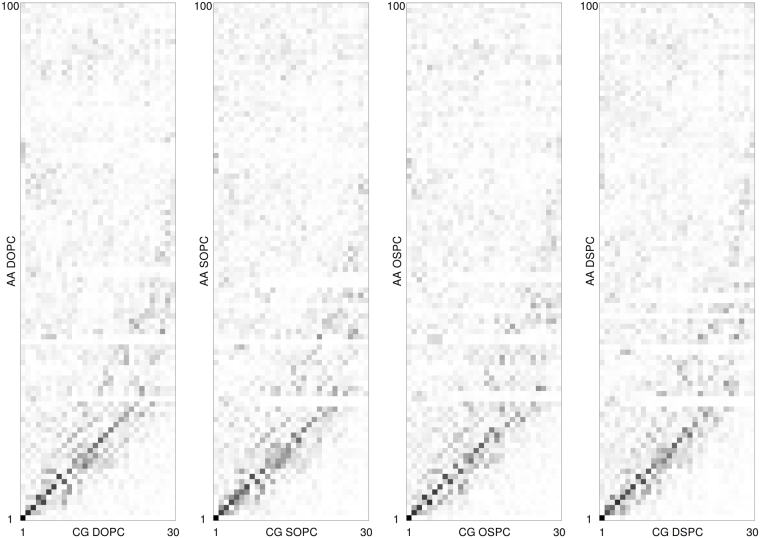
Comparison of the coarse grained PCA eigenvectors obtained in the AA simulations with the ones obtained in the CG simulations. Dot product matrices of the corresponding eigenvector sets are shown. Black and white correspond to the dot product equal to one and zero, respectively.

Next, we compared collective motions of each lipid at different resolution levels. Comparison of the coarse grained AA principal components with those from the CG simulations again reveals close correspondence and diagonal structure of the dot-product matrices, which are however significantly noisier, and non-major atomistic collective motions are not captured very well in the CG simulations (Fig. 4).

### General effects of reduction in dimensionality

Comparison of atomistic collective motions to those observed in CG simulations merits additional considerations. Development of each CG model comprises two major steps: 1) choice of the preferred CG model architecture and appropriate mapping; 2) parameterization of the constructed model^33, 34^. Since the number of degrees of freedom in CG models is always smaller than in atomic ones, some of atomic motions are inevitably eliminated. Therefore, part of the differences between the AA and CG simulations is inherent to the chosen architecture of the CG model and does not depend on its parameterization. We note that the loss of information associated with dimensionality reduction is usually not proportional to the number of degrees of freedom that are eliminated since the contribution and amplitudes of motions along different degrees of freedom are not equal. This is expected, because the goal of coarse graining is to reduce the computational complexity without losing the details of interest.

**Figure 5:**
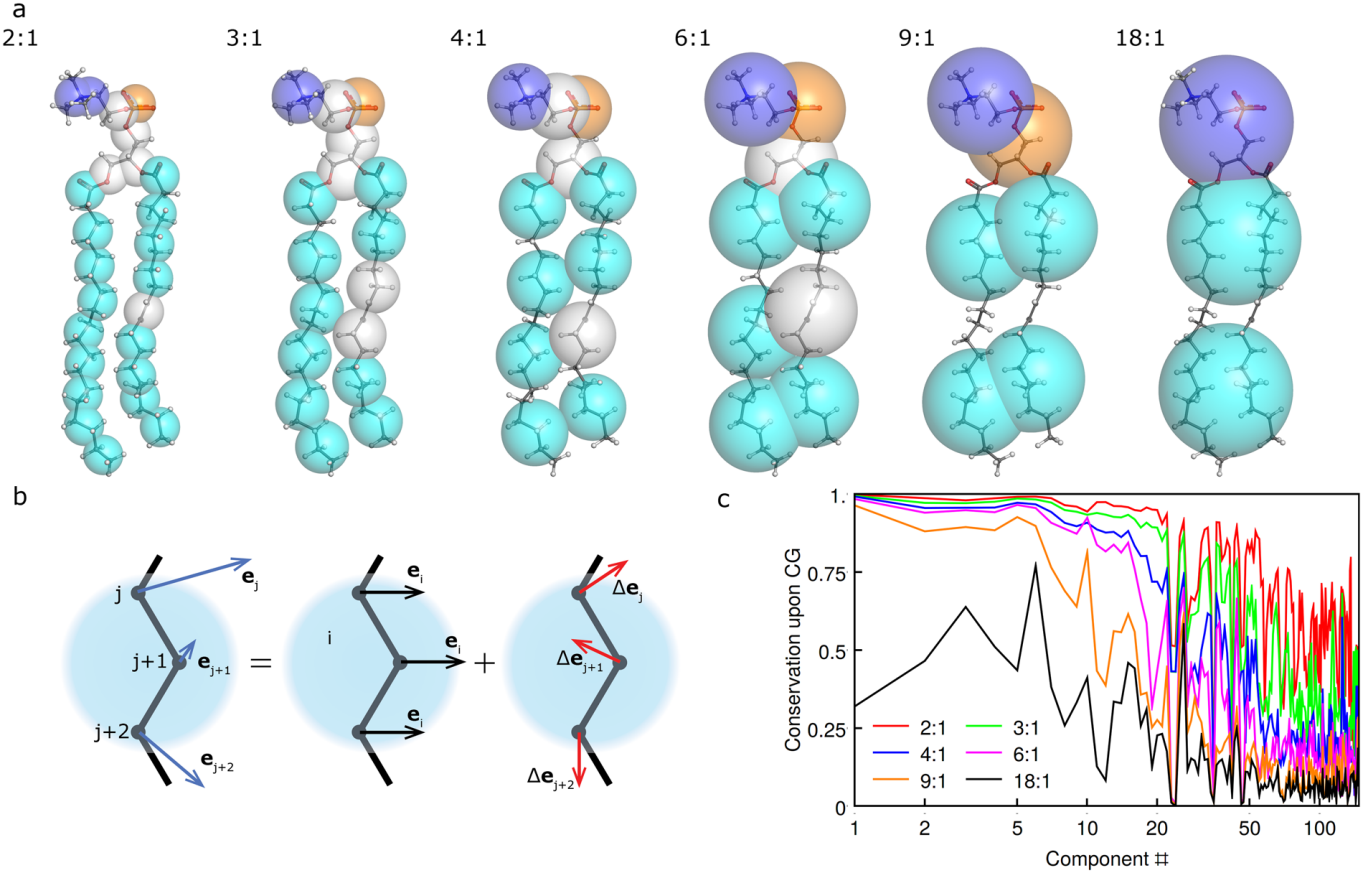
Loss of motion details upon coarse graining. (**a**) Hypothetical mappings with varying atom-to-bead ratios. Details of the mappings may be found in Supplementary Table S1. (**b**) Decomposition of atomic displacements into bead displacements and displacements of atoms relative to each other. (**c**) Conservation of SOPC collective motions (principal components) upon hypothetical coarse grainings using different atom-to-bead mappings.

To find how well the principal components are retained upon coarse graining, we analyze several hypothetical mappings with different atoms-to-beads ratios (Table S1, Fig. 5a). Displacement of each atom *j* in the atomic model can be represented as a displacement of the corresponding bead *i* in the CG model plus motion of the atom relative to the center of the bead (Fig. 5b). Motions of atoms relative to each other are eliminated in the CG model. The ratio of retained RMSD of the beads positions in the CG model to the RMSD of the atoms positions in the atomic model can be used to measure how well corresponding motion is conserved in the CG model. The ratio also should take into account different weights of different atoms in particular atom-to-bead mapping. With these considerations, we arrive at a formula described in Methods.

Recently, Foley *et al*. applied the relative entropy approach^73^ to evaluate the impact of CG mapping upon information in coarse-grained models^74^. We note that their approach is complementary to ours. Indeed, our approach provides the information about a particular motion irrespective of associated thermodynamic properties, whereas the relative entropy approach provides the thermodynamic information about the whole system (so-called mapping entropy) and relies on the chosen potential and thermodynamic properties of the reference (for example, atomistic) model^73, 74^.

Using SOPC as an example, we find that with the 2:1, 3:1, 4:1 and 6:1 mappings all of the major collective motions (up to PC18) are essentially conserved, whereas smaller amplitude motions are progressively eliminated (Fig. 5c). With the 9:1 mapping even the major PCs become affected. Finally, in the case of the extreme 18:1 coarse graining, similar to the model by Cooke *et al*.^75^, only the bending degrees of freedom are partially conserved, with other motions being almost completely eliminated (Fig. 5c). Overall, these results show that construction of meaningful CG models retaining most of the lipid collective motions is possible when up to ~6 heavy atoms are mapped to each CG bead.

**Figure 6:**
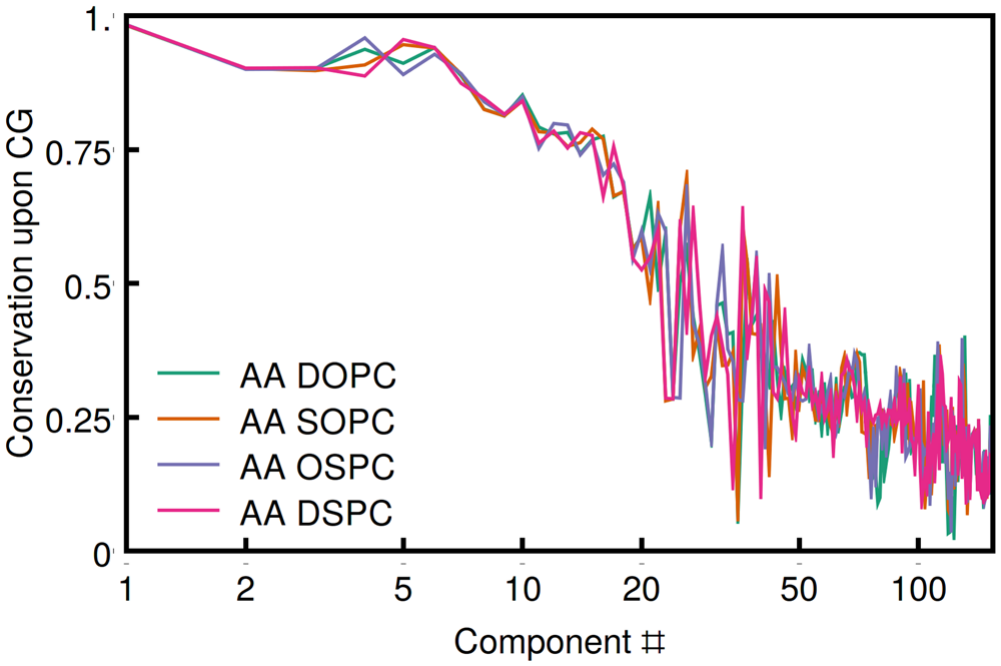
Conservation of the motions corresponding to different PCs in atomistic simulations upon Martini coarse graining (﻿please find the description in Methods). The major motions are very well conserved whereas the lower-amplitude PCs are mostly eliminated.

In Martini, each bead on average represents 4 heavy atoms^17^, however the bead definitions were recently updated to include up to 9 atoms with individual weights ranging from 1/13^th^ to 1/3^rd^ ^76^. We find that, similarly to the hypothetical CG models analyzed earlier, Martini representation retains 75% to 100% of the major DOPC, SOPC, OSPC and DPPC collective motions but significantly reduces the minor ones (Fig. 6). The degree of motion conservation upon coarse graining follows the same pattern as the collectivity of principal components (calculated previously^28^).

While principal components are orthogonal by definition, coarse graining and consequent reduction in dimensionality of the conformational space results in loss of some motions (displacements of the atoms within one CG bead relative to each other) and non-zero overlap of the remaining ones. Indeed, comparison of the coarse-grained AA principal components reveals almost complete loss of smaller-amplitude motions and emergence of spurious similarities between the larger-amplitude ones (Figure S8).

### Comparison of configurational spaces

**Figure 7:**
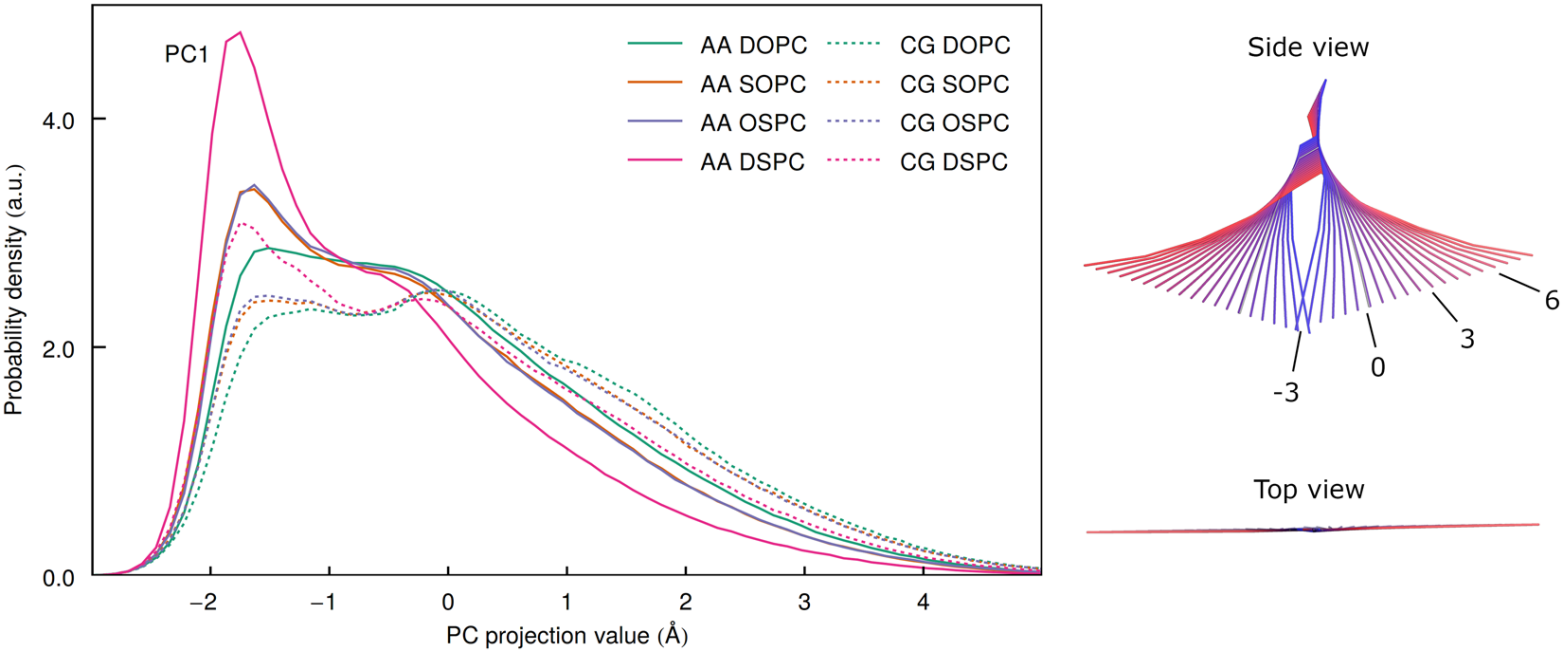
Influence of coarse graining and saturation of hydrocarbon chains on the scissoring motion of hydrophobic chains. Distributions of the PC1 projections in the same PCA eigenvector basis are shown on the left and corresponding structures are shown on the right.

**Figure 8:**
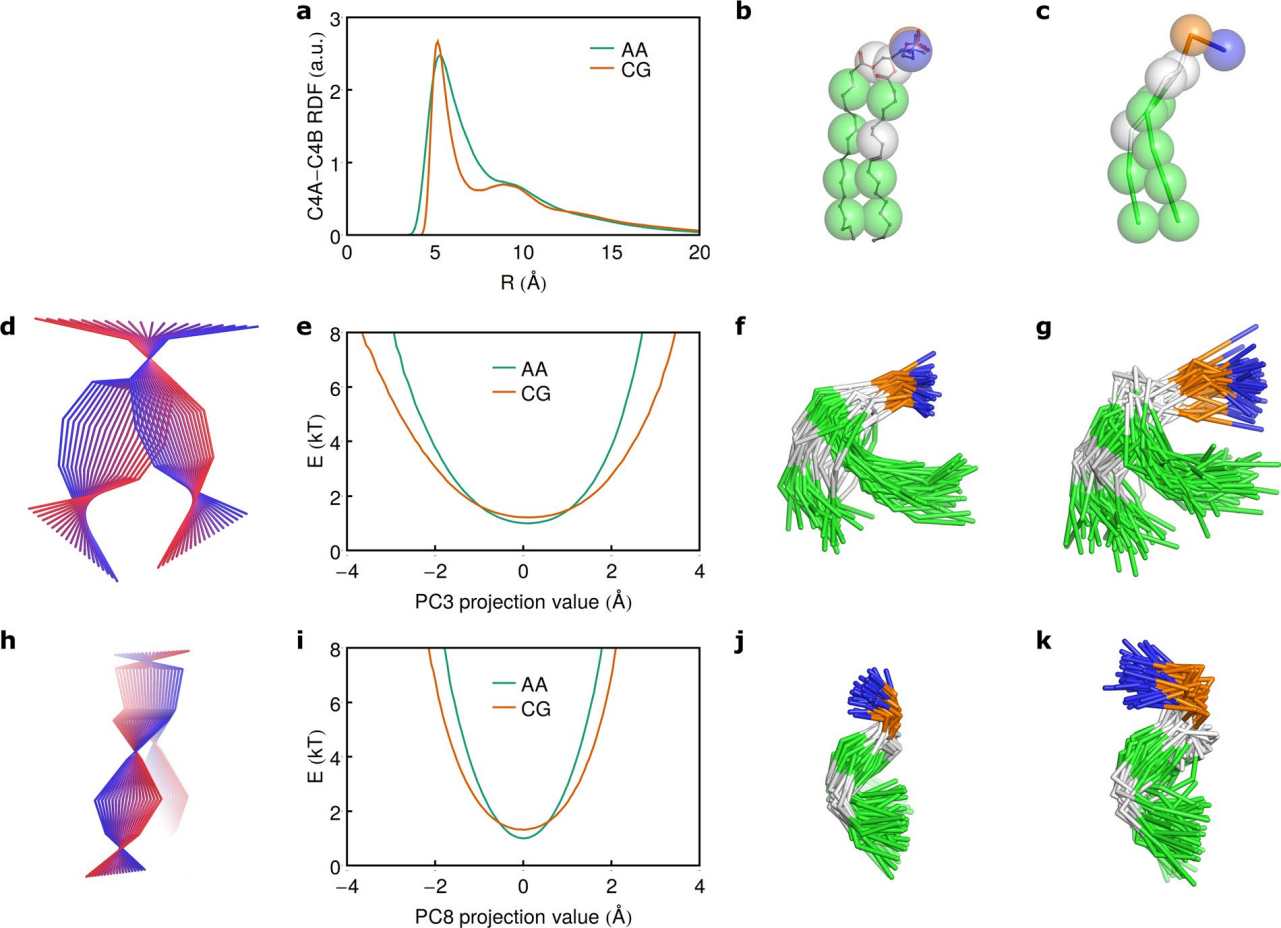
Examples of the extreme conformations from the AA and CG simulations, as exemplified by SOPC. (**a**) Radial distribution functions for the C4A and C4B beads belonging to the same molecule. AA simulations were coarse grained to obtain the data. (**b**,**c**) Conformations with the minimal C4A-C4B distances observed in AA and CG simulations (3.4 Å and 4.2 Å, respectively). In the atomic simulations, the inter-bead distance can become smaller due to intertwining of the hydrocarbon chains. (**d**) Collective motion associated with PC3. (**e**) Effective energy landscape for PC3. (**f**,**g**) Conformations with the smallest projection values on PC3 observed in AA and CG simulations (projection values from −3.5 to −3 Å and from −4.4 to −3.9 Å, respectively). In the CG simulations, the molecule is significantly more flexible. (**h**) Collective motion associated with PC8. (**i**) Effective energy landscape for PC8. (**j**,**k**) Conformations with the smallest projection on PC8 observed in AA and CG simulations (projection values from −2.2 to −1.9 Å and from −2.5 to −2.3 Å, respectively). In the CG simulations, the molecule is more flexible.

**Figure 9:**
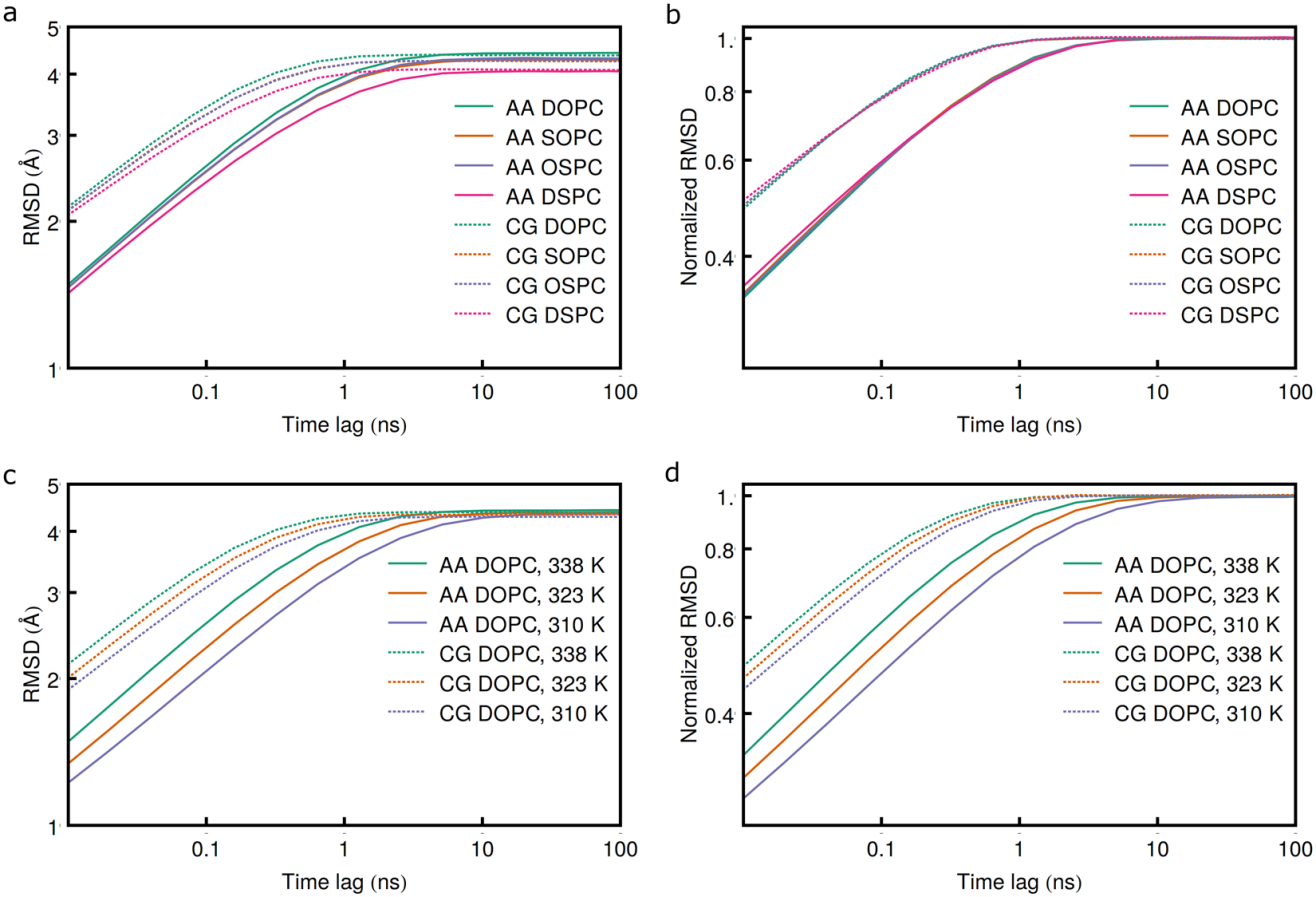
Average RMSD (left) and normalized RMSD (right) of lipid heavy atoms or particles positions as a function of time lag. Note that the structures were aligned prior to RMSD calculation and thus there is no contribution from diffusion.

Next, we analyze the conformational properties of the lipids by comparing the distributions of the PC projections in the common CG PCA basis (Figs 7 and 8 and S9–S10). As was observed before, the distribution of PC1 projection values is clearly non-Gaussian^28^. There are three peaks of varying amplitudes that correspond to the conformations of the lipid molecule where the hydrocarbon chains are in direct contact; separated by a single chain from another molecule; and separated by two and more other chains. Both in AA and CG simulations the first peak in the distribution is the highest for the DSPC, intermediate for SOPC/OSPC and the lowest for DOPC, and the distribution is the narrowest for DSPC, intermediate for SOPC/OSPC and widest for DOPC lipid molecules. The distributions for SOPC and OSPC are almost identical. Overall, this is in agreement with the observation that more unsaturated lipid molecules are more flexible and disordered^63^, and have higher equilibrium RMSD (Fig. 9). At the same time, for each lipid the first peak in the PC1 distribution is the highest in AA and the lowest in CG simulations, while the probability distribution is the narrowest in AA simulations and the widest in CG simulations. Perhaps, this is a consequence of higher number of interaction sites and resulting higher friction and restriction of motions in more detailed FFs^77^. Similar overestimation of flexibility in CG simulations was observed before^53, 55, 56^.

PC1 distributions prompted us to calculate intra- and interlipid radial distribution functions for the ends of hydrocarbon chains (Figures S11–12). The distributions of distances within one molecule (Figure S11) are understandably similar to distributions of projections on PC1 (Fig. 7). The first- and second-order RDF peaks in the CG simulations are much sharper (Figures S11–12). This has been noted before^53, 54^ and probably reflects the fact that CG chains cannot intertwine as easily as the chains in atomic representation. Indeed, comparison of the conformations with closest chain-end beads in atomic and CG simulations reveals exactly that (Fig. 8b,c). Similarly, other bead-bead RDFs in CG simulations might diverge from the ones in the atomic simulations, and thus CG potentials might need to be corrected as suggested elsewhere^40, 54, 78^.

The distributions for other PCs are closer to Gaussian, although some of them are clearly asymmetric (for example, PCs 5, 6, 7 and 10, Figure S9). Comparisons reveal that in many cases CG simulations result in much broader distributions both for in plane (for example, PC3, Fig. 8d) and out of plane (for example, PC8, Fig. 8h) motions. Evidently, lipid molecules in Martini simulations are much more flexible, and can access highly bent and possibly protruding conformations not accessible in atomic simulations (see examples in Fig. 8d–k). While no direct data on conformations of lipid molecules are available, single molecule protrusions have been observed both in simulations^79^ (the frequency of protrusions depends strongly on force field^21^) and fluorescence experiments with pyrene-labeled lipids^80, 81^. Still, the flexibility of the lipid molecules might be overestimated in CG simulations, as was observed before^53, 55, 56^.

### Effects of hydrocarbon chain saturation on conformations

Comparison of DOPC, SOPC, OSPC and DSPC configurational spaces in the same CG PCA basis reveals mostly similar distributions, with some collective motions revealing more differences (for example, PCs 1,5–10, Figure S9) than others (PCs 2–4, Figure S9).

Comparison of the lipids in atomistic PCA basis (Figures S13 and S14) reveals similar patterns. The differences and similarities in distributions depend on the nature of corresponding collective motion. PC1, which corresponds to scissoring motion (Figure S3), reveals more extended DOPC, intermediate and similar SOPC and OSPC, and more compact DSPC distributions (Figure S13). PC3, corresponding to mostly in-plane bending of the whole lipid molecule (Figure S3), reveals almost no differences between the lipids (Figure S13). On the contrary, PC6 is able to distinguish all the four lipids from each other (Figure S13).

Overall, the obtained results suggest that the effects of monounsaturated chains are additive. Pearson correlation coefficient (PCC) of the DOPC and SOPC/OSCP covariance matrices is the same as PCC of the SOPC/OSPC and DSPC covariance matrices, and PCC of the DOPC and DSPC covariance matrices is the same as PCC of the SOPC and OSPC covariance matrices. Distributions of SOPC and OSPC PC1 and most of other projections are similar, and are intermediate between those of DOPC and DPPC. At the same time, is should be noted that positional isomers of phosphatidylcholine lipids (such as SOPC and OSPC) have slightly different phase transition temperatures^82^, and thus contributions of the *sn*-1 and *sn*-2 chains to the lipids’ physical properties are not identical.

### Effects of temperature on conformations

Comparing AA and CG simulations of DOPC conducted at 310, 323 and 338 K, we observe very little differences in configurational spaces (Figures S15–S17). The lipids are slightly more ordered at lower temperatures (Figure S15), as could be expected, but no other significant effects of temperature on the available configurational space are observed. Covariance matrices show very little change with temperature both in AA and CG simulations (Figure S6).

### Convergence of the simulations

200 ns long trajectories of systems consisting of ~100 lipids with snapshots taken each 10 ps contain ~2·10^6^ individual conformations. As we discussed earlier, such simulation lengths are sufficient for relaxation of even the slowest motions and convergence of the observed variables^28^, with characteristic autocorrelation decay times converging to within 5% (data not shown).

We begin the analysis and comparison of characteristic time scales in the present simulations by studying the dependence of structure RMSD of individual lipid molecules on time lag (Fig. 9, note that the molecules were aligned prior to RMSD calculation, as described in Methods). Overall, coarse graining results in 4.6 (at 338 K) to 7.2 (at 310 K) times faster equilibration. DOPC RMSD values plateau at the highest levels, whereas SOPC/OSPC and DSPC RMSD values are intermediate and lowest, correspondingly. RMSD values for SOPC and OSPC are almost identical. At the same temperature DOPC, SOPC, OSPC and DSPC equilibrate at similar rates, however more unsaturated lipids equilibrate slightly faster. Fitting the normalized RMSD with exponential function ${c}_{1}{\tau }^{{c}_{2}}$ in the linear region of the plot reveals *c*_2_ values of 0.214, 0.207, 0.205 and 0.193 in the atomistic simulations, and 0.240, 0.233, 0.234 and 0.222 in the CG simulations, for DOPC, SOPC, OSPC and DSPC, respectively. Compared to the simulations conducted at 310 K, in atomistic simulations, RMSD converges 1.56 and 2.46 times faster at 323 K and 338 K, respectively, and in CG simulations, RMSD converges 1.26 and 1.59 times faster at 323 K and 338 K, respectively. The influence of temperature will be discussed below in more detail.

While studying RMSD time dependence is instructive, it does not provide definitive answers, because some particular motions may equilibrate much slower than the RMSD of the molecule as a whole^28^. Therefore, we investigated the dynamics and convergence of simulations in the coordinates defined by PCs.

### Characteristic equilibration time scales

**Figure 10:**
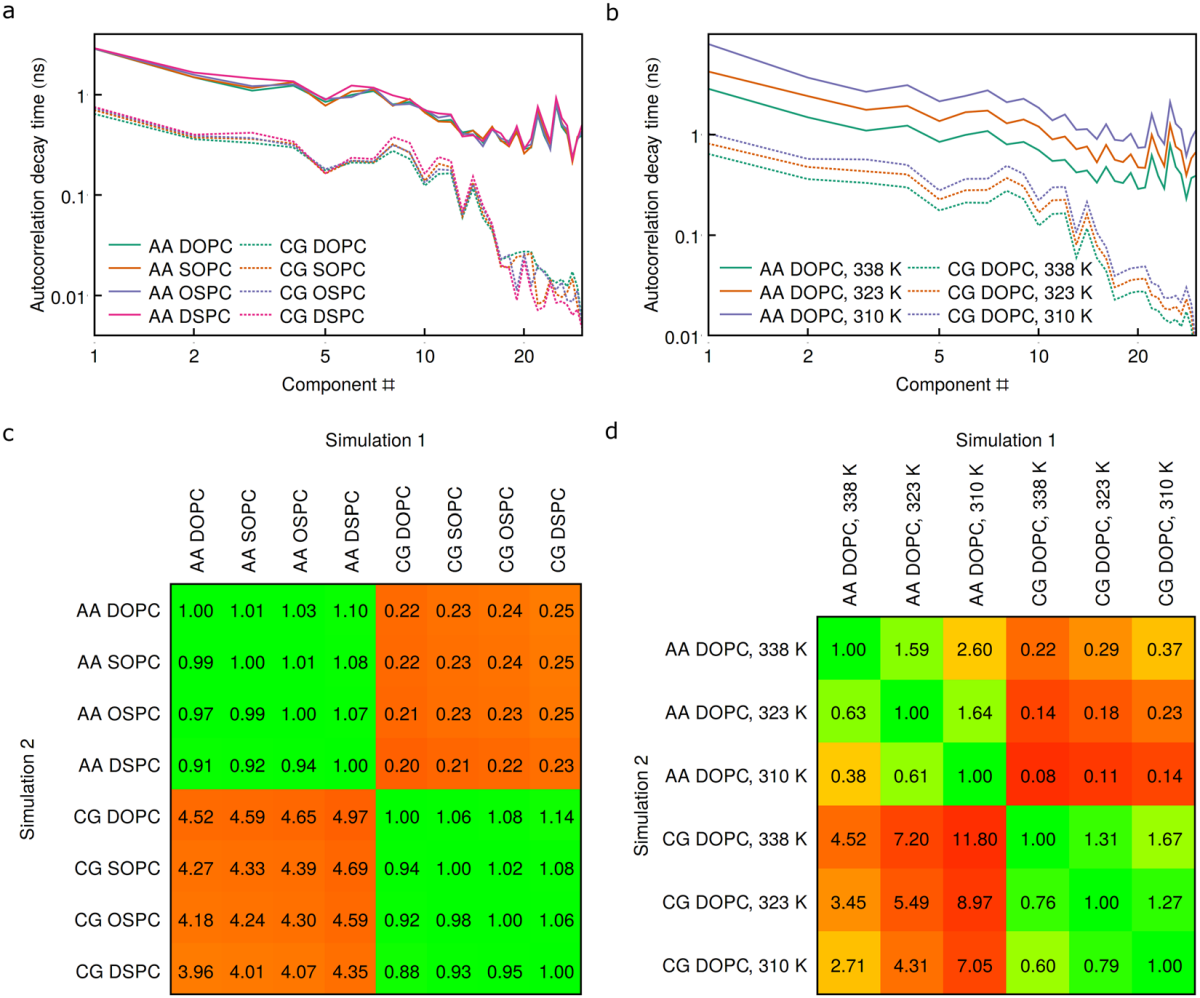
Characteristic autocorrelation decay times for the analysis in the joint PC basis, and corresponding speedups (geometric mean ratios, ﻿please find the description in Methods). The ratios are color-coded to highlight the similarities and dissimilarities between the simulations.

First of all, characteristic time scales (the time in which the autocorrelation decays in *e* ^2^ times) in different AA simulations conducted at the same temperature are very close to each other and are below 10 ns (Figs 10 and S18). As was observed for RMSD (Fig. 9), SOPC and OSPC equilibrate slightly slower than DOPC, and DSPC equilibrates notably slower. This slowdown is not uniform both in AA and CG simulations: for example, for PC1, DSPC is ~1% slower than DOPC in AA simulations (17% slower in CG); for PC3 DSPC is ~32% slower than DOPC in AA simulations (26% slower in CG). Similar trends are observed when PCA is conducted only on atomistic simulations in AA basis (Figure S19). Surprisingly, all of the collective motions are accelerated very homogenously with rising temperature (Figs 10 and S19).

In the CG simulations the autocorrelation decays ~4.5 times faster at 338 K, ~5.5 times faster at 323 K and ~7 times faster at 310 K, compared to AA simulations (Fig. 10). The speedup is roughly homogenous for the major principal components, but becomes more significant for lower-amplitude motions (Fig. 10), perhaps, due to filtering out of these motions in CG representation (Fig. 6). Therefore, the dynamical processes are accelerated unevenly in CG simulations, similarly to what was observed before^59^. The acceleration is different at different temperatures (Fig. 10), as has been observed for various thermodynamic properties^53^. The obtained values of ~7 fold acceleration at 310 K are slightly higher than what was observed previously, whereas 4–5 fold acceleration at 338 K is similar^19, 57, 59^.

## Conclusions

In this work, we studied the single molecule behavior of saturated and unsaturated lipids DOPC, SOPC, OSPC and DSPC in atomistic and CG MD simulations by means of principal components analysis. The analysis was effective at identification and pinpointing similarities and differences between the simulations. We found that the conformational spaces available to the four lipids significantly overlap, although there is a notable difference: lipids with more unsaturated hydrocarbon chains favor more extended conformations (Figs 2, 7 and 9). The dynamics of the four lipids is very similar when studied at the same temperature, with unsaturated lipids equilibrating slightly faster (Fig. 10). At lower temperatures, all of the motions are homogenously slowed down (Fig. 10). Finally, we demonstrated how the results of the AA simulations can be compared directly to the results of the CG simulations, and showed that the latter capture 75 to 100% of the major conformational changes (Figs 4–6). Our approach allowed us to identify the conformations that are observed in AA simulations and not observed in CG ones, and vice versa (Fig. 8). Overall, the sampling was ~4–7 times faster in the CG simulations (Fig. 10). The CG sampling speedups were roughly the same for all of the major motions, but increased for lower amplitude motions (Fig. 10a,b) that are weakly conserved in CG simulations (Figs 4–6). We expect that the results reported here will be useful for efficient quantitative comparisons of simulations conducted at different levels of resolution and for further development and improvement of Martini and other CG lipid force fields. Comparison of the average structures might be used to improve CG mappings, and comparison of covariance matrices, projection distributions and characteristic timescales might be used to optimize the restraints and force constants.

## Materials and Methods

### Performed simulations

Details of the performed simulations are summarized in Table 1. The simulations were performed using GROMACS version 5.1^83^. The simulations AA1-6 were performed using CHARMM36 force field and CHARMM TIP3P water model^84, 85^. The simulations CG1-6 were performed using Martini force field version 2.2^17–19^, and polarizable water model^86^ where 4 water molecules are treated as a polar solvent particle. 4-bead representation of oleic and stearic acids was used^87^. Initial structures and topologies for simulations AA6 and CG6 were prepared using the CHARMM-GUI Membrane Builder^88–92^. Initial structures for simulations AA1-5, CG1-5 were generated from the AA6 and CG6 initial structures. Unsaturated bonds were introduced into the structure and topology files manually where needed. AA and CG systems were energy-minimized and equilibrated for 50 ns preceding the production runs. Evolution of the areas per lipid in the simulated systems is shown in Figure S1.

### Simulation conditions

Parameters in the AA and CG simulations were chosen to match the original reports by J. Lee *et al*.^89^ and the recommended parameters from the Martini web site^76, 93^ (Martini_v2.x_common updated July 15^th^, 2015), respectively. In the AA and CG simulations the leapfrog integrator with the time step sizes of 2 fs and 20 fs, respectively, was used. The system snapshots were collected every 10 ps. Periodic boundary conditions were applied in all 3 directions. The centers of mass of the bilayer and solvent were fixed. In the AA simulations covalent bonds to hydrogen atoms were constrained using the SHAKE algorithm^94^. The simulations AA1,4-6, CG1,4-6 were conducted at the reference temperature of 338 K. The simulations AA2 and CG2 were conducted at 323 K, and AA3 and CG3 at 310 K. The simulations were conducted using the reference pressure of 1 bar. Lipid and water molecules were coupled to the temperature baths separately. The AA simulations were performed using Nosé-Hoover temperature coupling method^95^ with the coupling constant of 1 ps^−1^ and a semiisotropic Parrinello-Rahman barostat^96^ with the relaxation time of 5 ps. The CG simulations were performed with the velocity rescale thermostat^97^ with the coupling constant of 1 ps^−1^ and the semiisotropic Parrinello-Rahman barostat^96^ with the relaxation time of 12 ps.

The nonbonded pair list was updated every 20 steps with the cutoffs of 1.2 and 1.4 nm in the AA and CG simulations, respectively. Force-based switching function with the switching range of 1.0–1.2 nm and particle mesh Ewald (PME) method with 0.12 nm Fourier grid spacing and 1.2 nm cutoff were used for treatment of the van der Waals and electrostatic interactions in the AA simulations. Potentials shifted to zero at the cutoffs of 1.1 nm and a reaction-field potential with ${{\epsilon }}_{rf}=\infty$ were used for treatment of the van der Waals and electrostatic interactions in the CG simulations, as recommended^93^.

### Analysis

#### Structure alignment

All of the analyses of the lipid molecule conformations were preceded by non mass-weighted least squares alignment of the analyzed structures to the reference average structure (see Fig. 1c and d for the conformations from the simulations AA4 and CG4). The reference structure was obtained in two steps as described previously^28^. First, the structures from the simulation AA1 were aligned to an arbitrarily chosen structure, and the first average structure has been obtained. Second, the structures were realigned to the first average structure, resulting in the second average structure. The second average structure was not affected by the choice of the reference structure in the first step, and it was used as a reference structure for all other alignments. The AA structures were aligned using the heavy atoms. The CG structures were aligned to the coarse grained reference average structure. To highlight the differences in the positions of the hydrophobic chains, the average structures in the Fig. 2 were aligned to each other using the headgroup atoms and the first 8 atoms of the acyl chains (for AA simulations) and using the headgroup beads and the first 2 beads of the acyl chains (for CG simulations).

#### Coarse graining of atomistic structures

Atomic structures were coarse grained using *backward* ^98^:

1 $${\rm{C}}={\omega }_{ij}{\rm{A}},$$

where A is a *n* × 3 matrix of atomistic coordinates in Cartesian space, C is a *m* × 3 matrix of coordinates of coarse grained particles, and *ω*_*ij*_ is the CG mapping matrix. The mappings were based on the data from the Martini web site^76^. Atomic displacements, corresponding to principal components, were obtained similarly:

2 $${{\boldsymbol{e}}}_{i}^{cg}={\sum }_{j=1}^{n}{\omega }_{ij}{{\boldsymbol{e}}}_{j}^{aa},$$

where ${{\boldsymbol{e}}}_{i}^{aa}$ is the vector in the atomistic representation and ${{\boldsymbol{e}}}_{i}^{cg}$ is the corresponding vector in the CG space.

#### Pearson correlation coefficient

Pearson correlation coefficients of the covariance matrices *M* ^*i*^ and *M* ^*j*^ obtained in simulations *i* and *j*, correspondingly, were calculated as follows:

3 $${r}_{ij}=\frac{{\rm{cov}}({M}^{i},{M}^{j})}{\sigma {M}^{i}\cdot \sigma {M}^{j}}$$

where cov(*M* ^*i*^, *M* ^*j*^) is the covariance of the elements of *M* ^*i*^ and *M* ^*j*^, and *σM* ^*i*^ and *σM* ^*j*^ are the standard deviations of the elements of *M* ^*i*^ and *M* ^*j*^. The covariance matrices for calculation of the Pearson correlation coefficients were obtained using the coarse grained trajectories (Figs 3, S4–S6 and S19).

#### Principal component analysis

In AA representation, PCA was performed on the positions of heavy atoms. In the CG representations, PCA was performed on the positions of the beads. PCA^72^ was performed using the *covar* and *anaeig* utilities of the GROMACS software package^83^ as described previously^28^. *covar* was used to calculate the atomic displacement covariance matrices, their eigenvalues and eigenvectors, and *anaeig* was used to project the molecular dynamics trajectories onto the obtained eigenvector bases. Three types of analyses were performed. First, PCA was performed for each simulation separately (Figs 3, 4, 6 and S2–S8). Second, PCA was performed jointly for simulations AA1-6 and CG1-6 in the CG representation (Figs 7, 8, 10, S9–S10 and S15–S18). Finally, PCA was performed jointly for AA1-6 in the AA representation (Figs S13–S14 and S19). For comparisons of the results of the simulations for different lipids and topologies the eigenvalues were normalized by dividing by the number of particles in the model. Conformational spaces were compared using dot product matrices of eigenvectors mapped to the CG space (Figs 4 and S8) or eigenvectors in the native AA or CG basis (Figure S7).

#### Conservation upon coarse graining

Conservation of PCA eigenvectors upon coarse graining (Figs 5c and 6) was calculated as follows:

4 $$\alpha =\frac{{{\rm{RMSD}}}^{cg}}{{{\rm{RMSD}}}^{aa}}=\frac{\sqrt{\frac{1}{m}{\sum }_{i=1}^{m}{\sum }_{j=1}^{n}{\omega }_{ij}{{\boldsymbol{e}}}_{ji}^{aa\_c{g}^{2}}}}{\sqrt{\frac{1}{m}{\sum }_{i=1}^{m}{\sum }_{j=1}^{n}{\omega }_{ij}{{\boldsymbol{e}}}_{j}^{a{a}^{2}}}}=\frac{\sqrt{\frac{1}{m}{\sum }_{i=1}^{m}{{\boldsymbol{e}}}_{i}^{c{g}^{2}}}}{\sqrt{\frac{1}{m}{\sum }_{i=1}^{m}{\sum }_{j=1}^{n}{\omega }_{ij}{{\boldsymbol{e}}}_{j}^{a{a}^{2}}}}\le 1$$

where ${{\boldsymbol{e}}}_{i}^{aa}$ is the *n*-dimensional vector of atomic displacements in the all atom representation, ${{\boldsymbol{e}}}_{ji}^{aa\_cg}$ is the *n*-dimensional vector of atomic displacements, where displacement of each atom *j* is the same as displacement of the corresponding bead *i*, ${{\boldsymbol{e}}}_{i}^{cg}$ is the *m*-dimensional vector of bead displacements upon the coarse graining procedure, and *ω*_*ij*_ is the CG mapping matrix. Eliminated atomic motions ${{\boldsymbol{e}}}_{j}^{el}\,\,$constitute the kernel (null space) of the CG mapping matrix:

5 $${\sum }_{j=1}^{n}{\omega }_{ij}{{\boldsymbol{e}}}_{j}^{el}=0$$

#### Radial distribution function

The radial distribution functions were calculated using the *rdf* utility of the GROMACS software package^83^.

#### Effective potentials

Effective potentials associated with the conformational changes along the eigenvectors were calculated as follows (Figs 8e,i, S9–S10 and S13, S14, S16 and S17):

6 $$E(x)=-{k}_{B}T\,\mathrm{log}\,P(x)$$

where *P*(*x*) is the probability density and *x* is the value of the projection on corresponding PC eigenvector.

#### Root-mean-square-deviation

Root-mean-square deviations (RMSD), presented in Fig. 9, were calculated as follows:

7 $${\rm{RMSD}}(\tau )=\frac{1}{{N}_{L}}\sum _{i=1}^{{N}_{L}}{\langle {{\rm{RMSD}}}_{i}(t,t+\tau )\rangle }_{t}$$

where *N*_*L*_ is the number of the lipid molecules present in the system, and 〈*RMSD*_*i*_(*t*, *t* + *τ*)〉_*t*_ is the root-mean-square deviation of the *i*-th molecule heavy atoms positions between the time moments *t* and *t* + *τ*, averaged over the time *t*. Normalized RMSD (NRMSD) was calculated as follows:

8 $${\rm{NRMSD}}(\tau )=\frac{{\rm{RMSD}}(\tau )}{{{\rm{RMSD}}}_{eq}}$$

where RMSD_*eq*_ is the equilibrium value of RMSD at *τ* → ∞. NRMSD was fitted with exponential function in the linear region of the plot (normalized RMSD values below 0.7):

9 $${\rm{NRMSD}}(\tau )={c}_{1}{\tau }^{{c}_{2}}$$

#### Autocorrelation

Autocorrelation *R* of the projections on principal components was calculated as follows:

10 $$R(\tau )=\frac{{\langle (p(t)-\langle p(t)\rangle }_{t})(p(t+\tau )-{\langle p(t){\rangle }_{t})\rangle }_{t}}{{\langle {(p(t)-{\langle p(t)\rangle }_{t})}^{2}\rangle }_{t}}$$

where *p*(*t*) is the projection value at the time *t* and 〈•〉_*t*_ is averaging over *t*.

#### Characteristic time scales

As autocorrelation decay could not be adequately fitted with power or exponential function^28^, we calculated characteristic times *τ* needed for autocorrelation to decrease in *e* ^2^ times from the starting value, where *e* is the natural logarithm base (Figs 10 and S18–S19). For simplicity, the starting value for autocorrelation was assumed to be 1.0. The time dependence values were linearly extrapolated where needed.

The eigenvalue-weighted geometric means of the characteristic time scale ratios in the simulations *i* and *j*, measured in common PC basis, were calculated as follows:

11 $$R(i,j)=\exp (\sum _{k=1}^{{N}_{F}}\beta {E}_{k}\,\mathrm{log}\,\frac{{\tau }_{k}^{i}}{{\tau }_{k}^{j}})$$

where *N*_*F*_ is the number of the degrees of freedom for lipid model, *E*_*k*_ is the *k*-th eigenvalue of the covariance matrix, *β* is the normalization factor chosen so that ${\sum }_{k=1}^{{N}_{F}}\beta {E}_{k}=1$, and ${\tau }_{k}^{i}$ and ${\tau }_{k}^{j}$ are the characteristic time scales of the distributions convergence or autocorrelation decay for the *k*-th PC in the simulations *i* and *j*.

## Electronic supplementary material

Supplementary Information
