# Supplementary material for: Effects of Coarse Graining and Saturation of Hydrocarbon Chains on Structure and Dynamics of Simulated Lipid Molecules
Source: Sci Rep. 2017 Sep 13;7:11476. doi: 10.1038/s41598-017-11761-5 (PMC5597592; doi:10.1038/s41598-017-11761-5)
Supplement: Supplementary file 1 — Supplementary Information [file 41598_2017_11761_MOESM1_ESM.pdf]

# Effects of Coarse Graining and Saturation of Hydrocarbon Chains on Structure and Dynamics of Simulated Lipid Molecules

Pavel Buslaev* and Ivan Gushchin*

*E-mail for correspondence: pbuslaev@phystech.edu, ivan.gushchin@phystech.edu

Supplementary materials:

Supplementary Table S1

Supplementary Figures S1-S19

**Supplementary Table 1.** Hypothetical SOPC CG mappings used for calculations in Figure 5.

| CHARMM36 atom name | 2:1 bead | 3:1 bead | 4:1 bead | 6:1 bead | 9:1 bead | 18:1 bead |
|---|---|---|---|---|---|---|
| C13 | 1 | 1 | 1 | 1 | 1 | 1 |
| C14 | 1 | 1 | 1 | 1 | 1 | 1 |
| C15 | 1 | 1 | 1 | 1 | 1 | 1 |
| N | 2 | 1 | 1 | 1 | 1 | 1 |
| C12 | 2 | 2 | 2 | 1 | 1 | 1 |
| C11 | 3 | 2 | 2 | 1 | 1 | 1 |
| O12 | 3 | 2 | 2 | 2 | 1 | 1 |
| P | 4 | 3 | 3 | 2 | 2 | 1 |
| O13 | 4 | 3 | 3 | 2 | 2 | 1 |
| O14 | 4 | 3 | 3 | 2 | 2 | 1 |
| O11 | 5 | 4 | 3 | 2 | 2 | 1 |
| C1 | 5 | 4 | 4 | 3 | 2 | 1 |
| C2 | 6 | 4 | 4 | 3 | 2 | 1 |
| O21 | 6 | 5 | 4 | 3 | 2 | 1 |
| C3 | 7 | 5 | 4 | 3 | 2 | 1 |
| O31 | 7 | 5 | 4 | 3 | 2 | 1 |
| C21 | 8 | 6 | 5 | 4 | 3 | 2 |
| O22 | 8 | 6 | 5 | 4 | 3 | 2 |
| C22 | 8 | 6 | 5 | 4 | 3 | 2 |
| C23 | 9 | 6 | 5 | 4 | 3 | 2 |
| C24 | 9 | 7 | 5 | 4 | 3 | 2 |
| C25 | 10 | 7 | 6 | 4 | 3 | 2 |
| C26 | 10 | 7 | 6 | 4 | 3 | 2 |
| C27 | 11 | 8 | 6 | 5 | 3 | 2 |
| C28 | 11 | 8 | 6 | 5 | 3 | 2 |
| C29 | 12 | 8 | 7 | 5 | 3 | 2 |
| C210 | 12 | 9 | 7 | 5 | 4 | 3 |
| C211 | 13 | 9 | 7 | 5 | 4 | 3 |
| C212 | 13 | 9 | 7 | 5 | 4 | 3 |
| C213 | 14 | 10 | 7 | 6 | 4 | 3 |
| C214 | 14 | 10 | 8 | 6 | 4 | 3 |
| C215 | 15 | 10 | 8 | 6 | 4 | 3 |
| C216 | 15 | 11 | 8 | 6 | 4 | 3 |
| C217 | 16 | 11 | 8 | 6 | 4 | 3 |
| C218 | 16 | 11 | 8 | 6 | 4 | 3 |
| C31 | 17 | 12 | 9 | 7 | 5 | 2 |
| O32 | 17 | 12 | 9 | 7 | 5 | 2 |
| C32 | 17 | 12 | 9 | 7 | 5 | 2 |
| C33 | 18 | 12 | 9 | 7 | 5 | 2 |
| C34 | 18 | 13 | 9 | 7 | 5 | 2 |
| C35 | 19 | 13 | 10 | 7 | 5 | 2 |
| C36 | 19 | 13 | 10 | 7 | 5 | 2 |
| C37 | 20 | 14 | 10 | 8 | 5 | 2 |
| C38 | 20 | 14 | 10 | 8 | 5 | 2 |
| C39 | 21 | 14 | 11 | 8 | 5 | 2 |
| C310 | 21 | 15 | 11 | 8 | 6 | 3 |
| C311 | 22 | 15 | 11 | 8 | 6 | 3 |
| C312 | 22 | 15 | 11 | 8 | 6 | 3 |
| C313 | 23 | 16 | 11 | 9 | 6 | 3 |
| C314 | 23 | 16 | 12 | 9 | 6 | 3 |
| C315 | 24 | 16 | 12 | 9 | 6 | 3 |
| C316 | 24 | 17 | 12 | 9 | 6 | 3 |
| C317 | 25 | 17 | 12 | 9 | 6 | 3 |
| C318 | 25 | 17 | 12 | 9 | 6 | 3 |

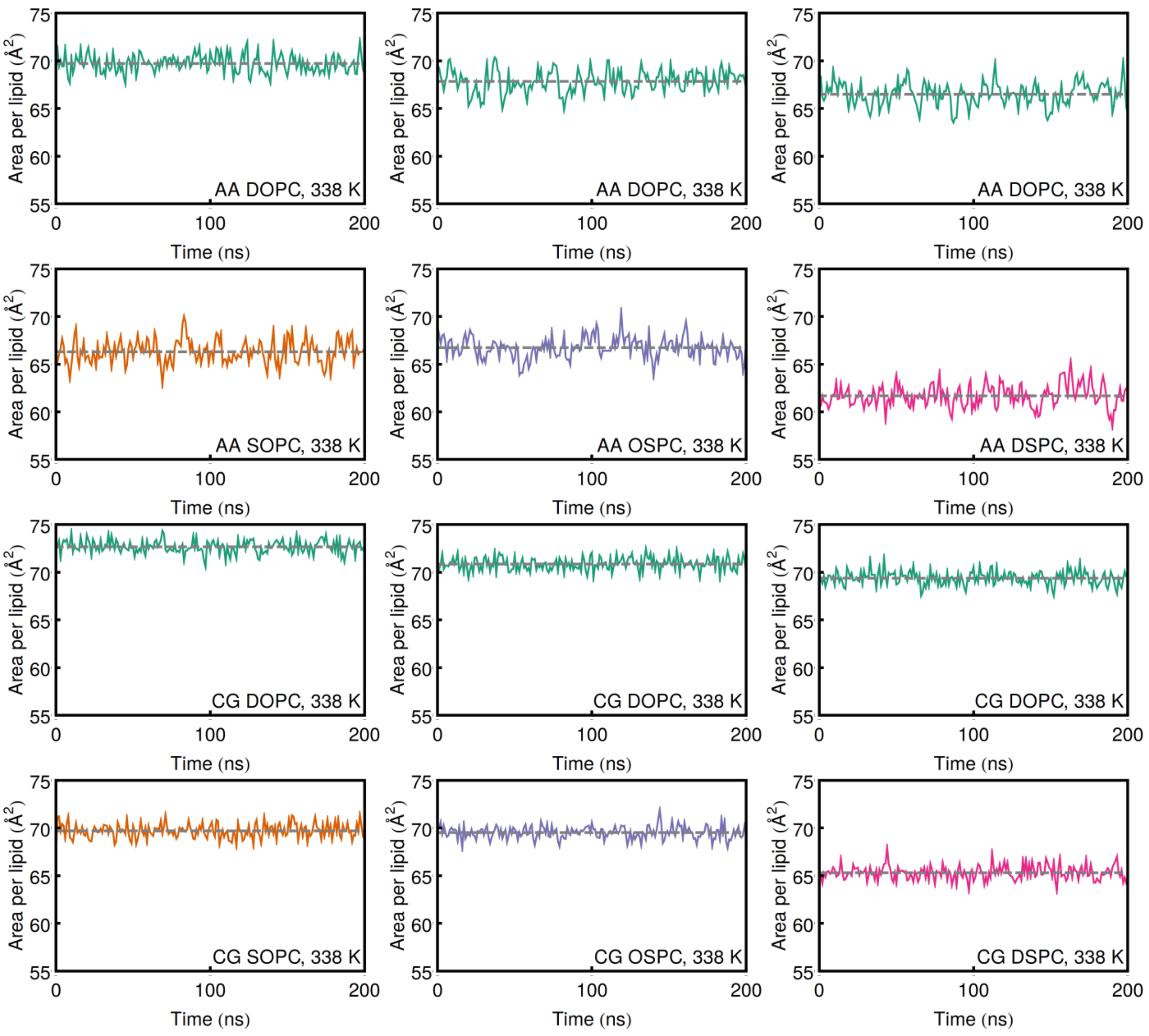


**Figure S1.** Evolution of the areas per lipid for the studied systems. The values are averaged over the time intervals of 1 ns. Total averages are shown with the gray dashes.

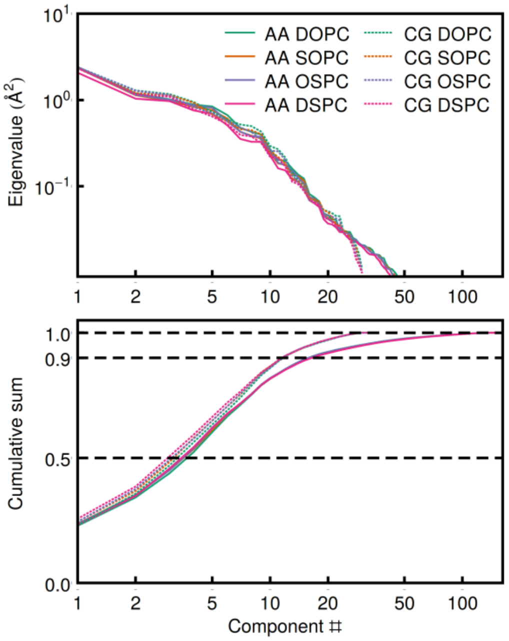


**Figure S2.** Principal component analysis of the lipid molecule conformations. (top) Eigenvalues of the covariance matrix of atomic displacements. Eigenvalues were normalized to the number of atoms in the corresponding model. (bottom) Cumulative sum of the eigenvalues, normalized to 1. Four main components account for ~50% of the structural variation, and 90% of structural variation are covered by 14 components in the AA simulations, and by 11 components in the CG simulations.

**Figure S3 (next page).** Conformational changes, associated with the top 10 principal components in each simulation in its own basis, and in common AA and CG bases (side and top views). Structures are colored from blue to red according to the PC projection value.

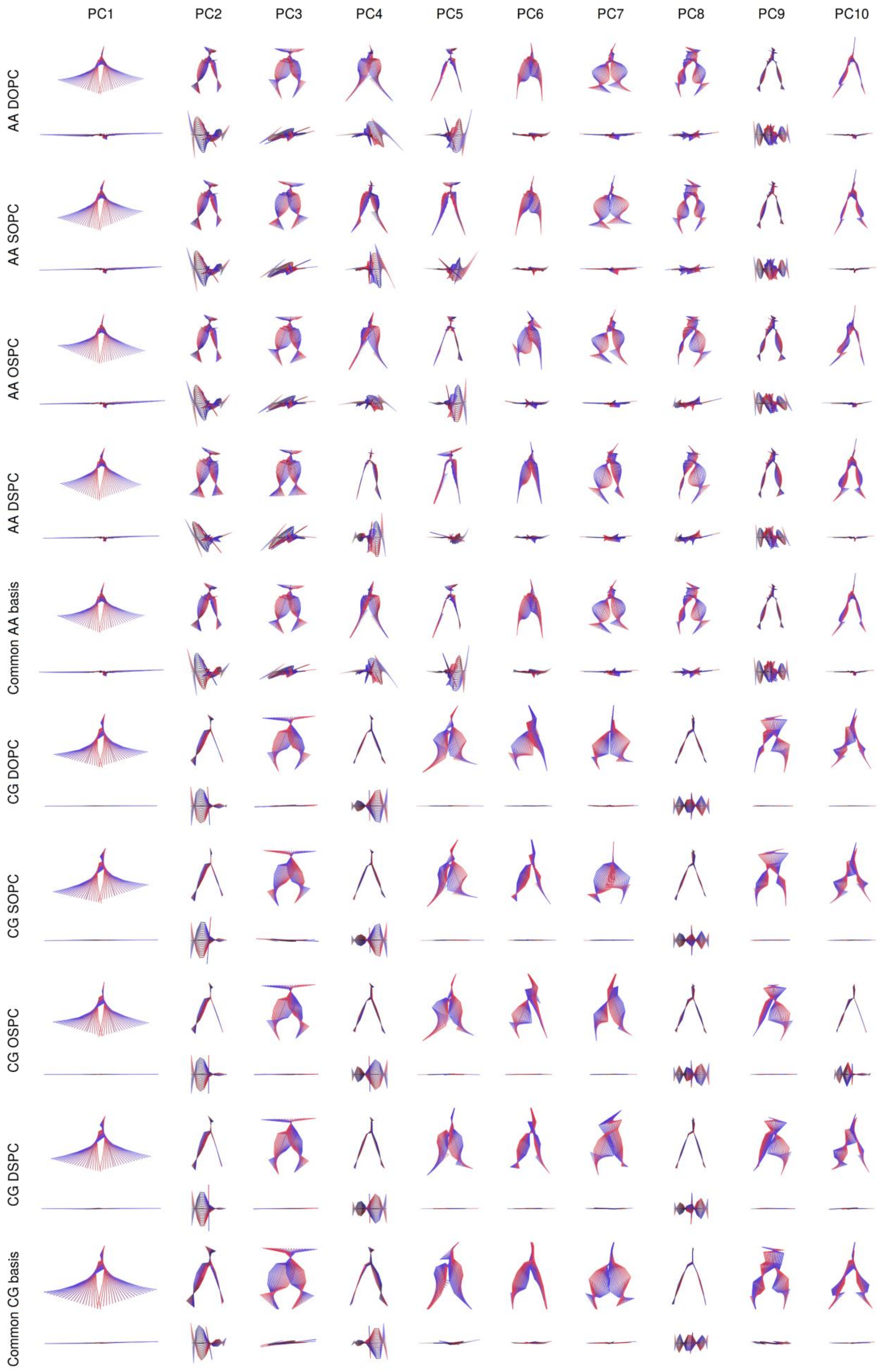
PC1
PC2
PC3
PC4
PC5
PC6
PC7
PC8
PC9
PC10
AA DOPC
AA SOPC
AA OSPC
AA DSPC
Common AA basis
CG DOPC
CG SOPC
CG OSPC
CG DSPC
Common CG basis

| | AA DOPC, 338 K | AA SOPC, 338 K | AA OSPC, 338 K | AA DSPC, 338 K |
|---|---|---|---|---|
| AA DOPC, 338 K | 1.000 | 0.996 | 0.996 | 0.991 |
| AA SOPC, 338 K | 0.996 | 1.000 | 0.991 | 0.994 |
| AA OSPC, 338 K | 0.996 | 0.991 | 1.000 | 0.994 |
| AA DSPC, 338 K | 0.991 | 0.994 | 0.994 | 1.000 |

**Figure S4.** Pearson correlation coefficients between the atomistic covariance matrices of DOPC, SOPC, OSPC and DSPC. The correlation coefficients are color-coded to highlight the similarity and dissimilarity between the simulations, as in other figures.

| | GAFF | Lipid14 | Slipids | CHARMM36 | CHARMM27 | GROMOS43 | GROMOS54 | Berger |
|---|---|---|---|---|---|---|---|---|
| AA DOPC, 310 K | 0.981 | 0.989 | 0.989 | 0.995 | 0.982 | 0.972 | 0.971 | 0.964 |
| CG DOPC, 310 K | 0.938 | 0.946 | 0.944 | 0.943 | 0.919 | 0.927 | 0.922 | 0.911 |

**Figure S5.** Pearson correlation coefficients between the covariance matrices of the present DOPC simulations (at 310 K) and those performed in our previous work (also at 310 K). The correlation coefficients are color-coded to highlight the similarity and dissimilarity between the simulations.

| | AA DOPC, 338 K | AA DOPC, 323 K | AA DOPC, 310 K | CG DOPC, 338 K | CG DOPC, 323 K | CG DOPC, 310 K |
|---|---|---|---|---|---|---|
| AA DOPC, 338 K | 1.000 | 1.000 | 0.999 | 0.956 | 0.952 | 0.952 |
| AA DOPC, 323 K | 1.000 | 1.000 | 1.000 | 0.956 | 0.952 | 0.952 |
| AA DOPC, 310 K | 0.999 | 1.000 | 1.000 | 0.955 | 0.951 | 0.951 |
| CG DOPC, 338 K | 0.956 | 0.956 | 0.955 | 1.000 | 1.000 | 1.000 |
| CG DOPC, 323 K | 0.954 | 0.953 | 0.953 | 1.000 | 1.000 | 1.000 |
| CG DOPC, 310 K | 0.952 | 0.952 | 0.951 | 1.000 | 1.000 | 1.000 |

**Figure S6.** Pearson correlation coefficients between the covariance matrices of the DOPC simulations conducted at different temperatures. The correlation coefficients are color-coded to highlight the similarity and dissimilarity between the simulations.

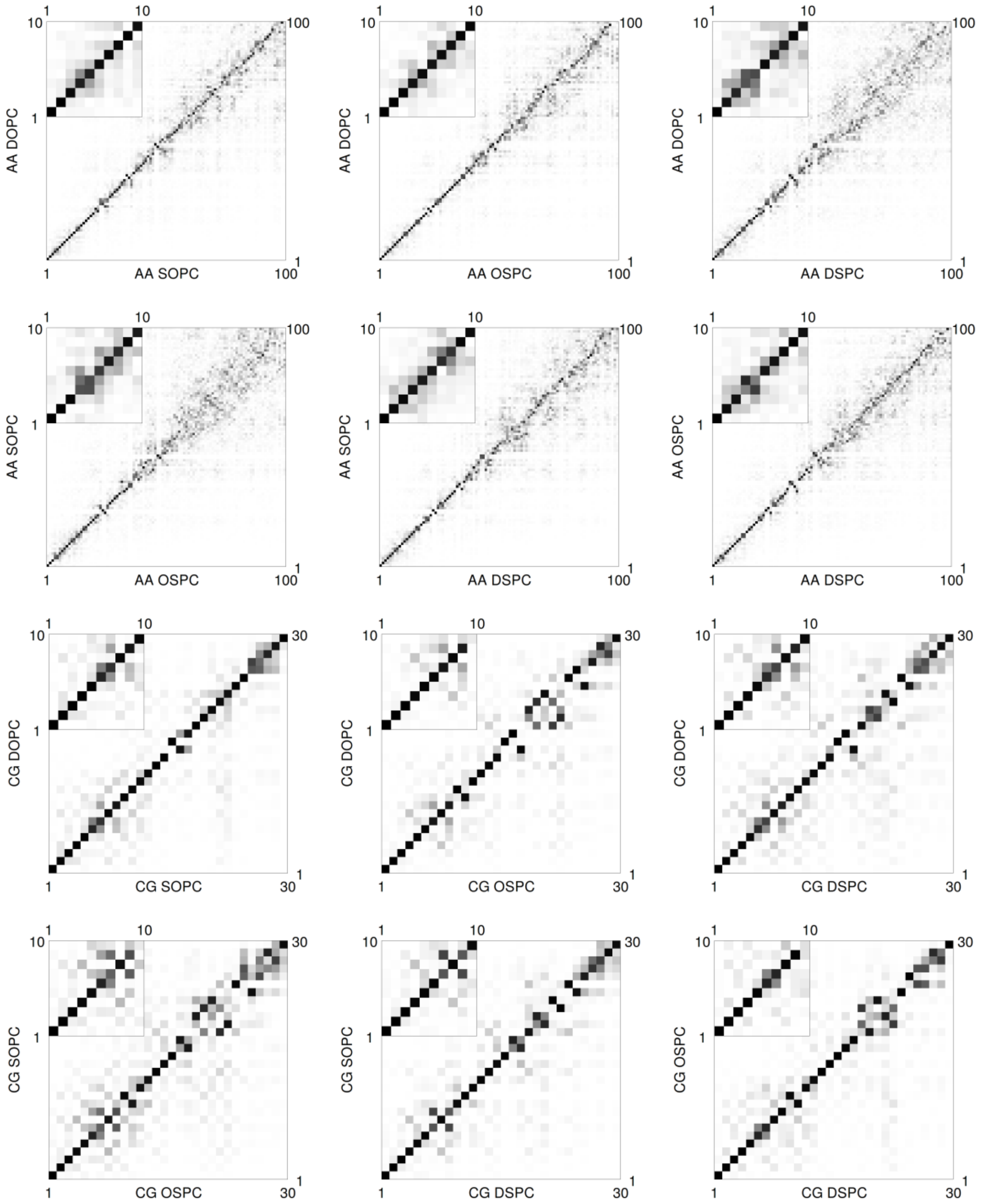


**Figure S7.** Comparison of PCA eigenvectors obtained in different simulations. The trajectories were coarse grained prior to PCA. Dot product matrices of the corresponding eigenvector sets are shown. The matrices have roughly diagonal structures, highlighting the similarity of the conformational spaces of DOPC, SOPC, OSPC and DSPC.

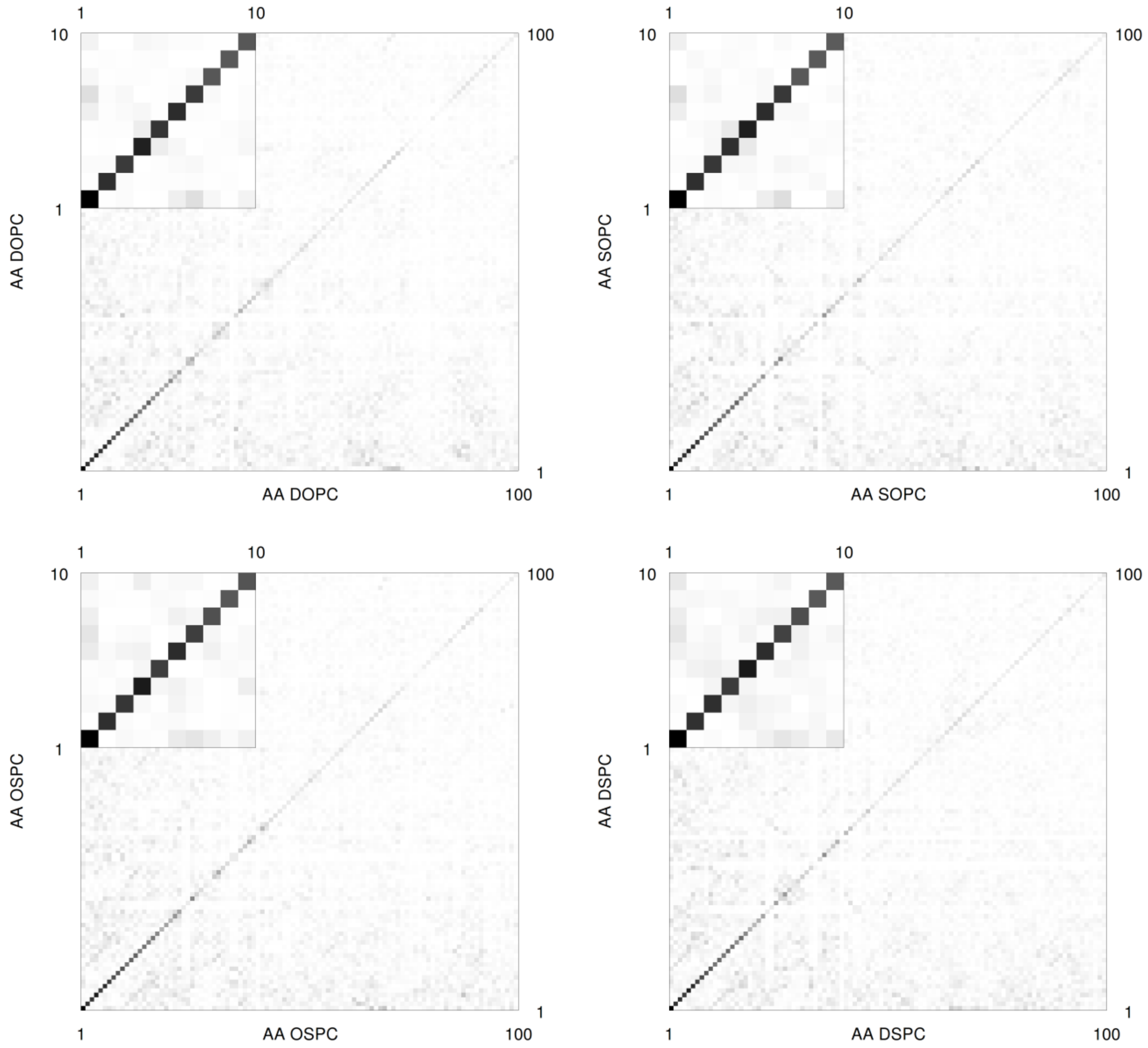


**Figure S8.** Effects of coarse graining on PCA eigenvectors obtained in the AA simulations. Dot product matrices are shown. The coarse grained eigenvectors are neither normalized nor orthogonal to each other after coarse graining.

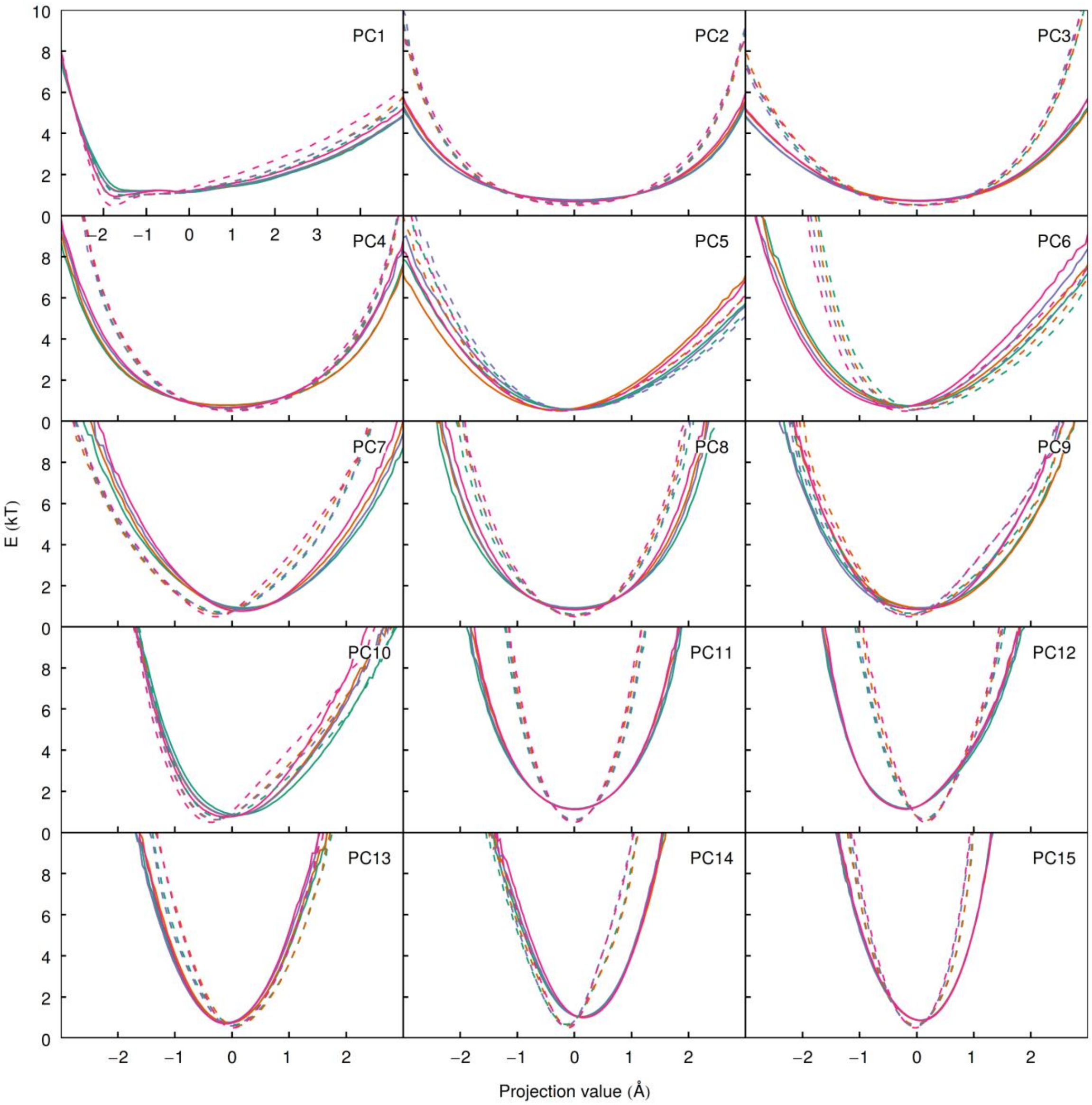


**Figure S9.** Effective potentials associated with the conformational changes along PC1–PC15. Data for DOPC, SOPC, OSPC and DSPC are in marine, orange, violet and magenta, respectively. Data for AA and CG simulations are depicted with solid and dashed lines, respectively.

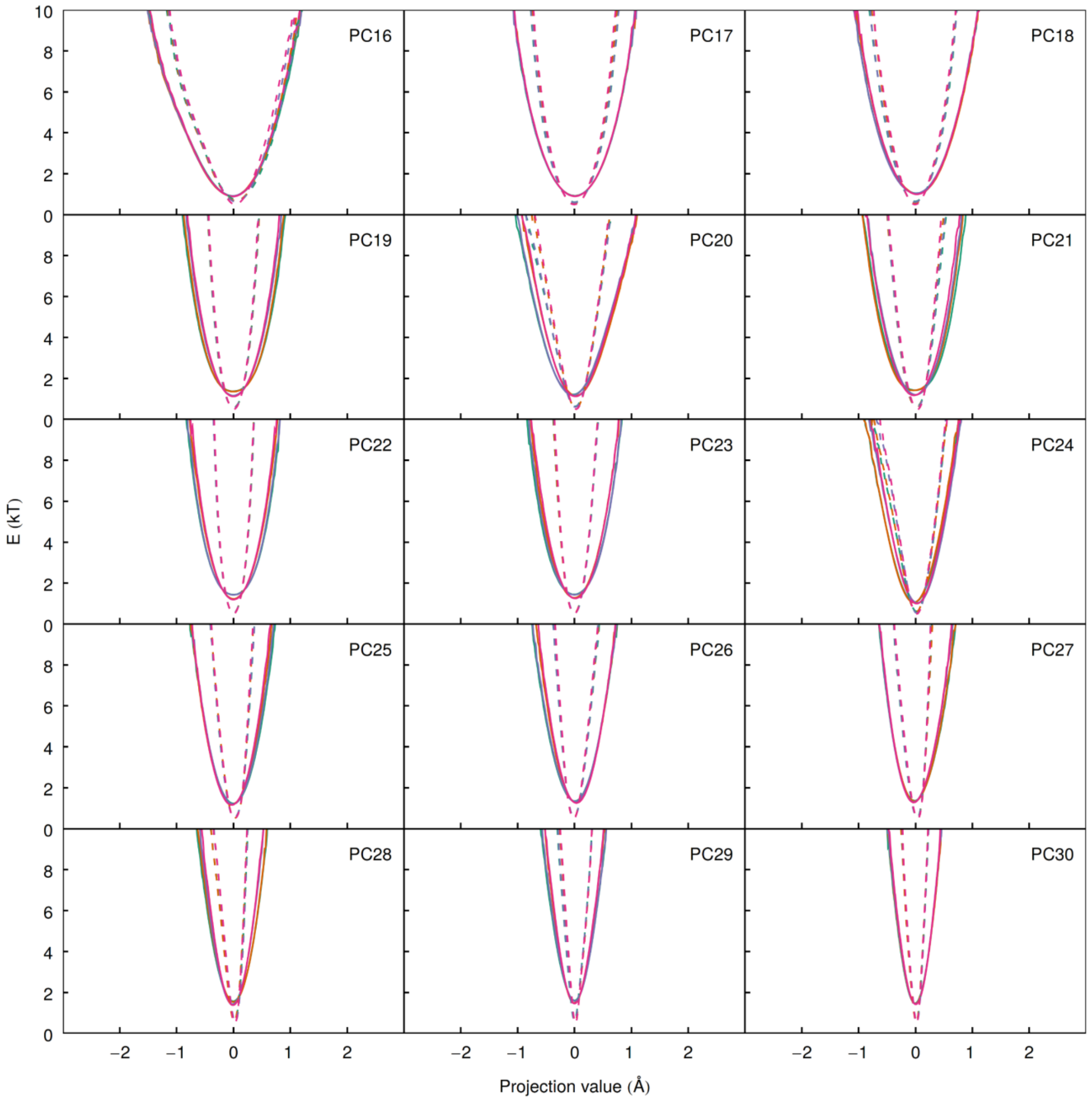


**Figure S10.** Effective potentials associated with the conformational changes along PC16–PC30. Data for DOPC, SOPC, OSPC and DSPC are in marine, orange, violet and magenta, respectively. Data for AA and CG simulations are depicted with solid and dashed lines, respectively.

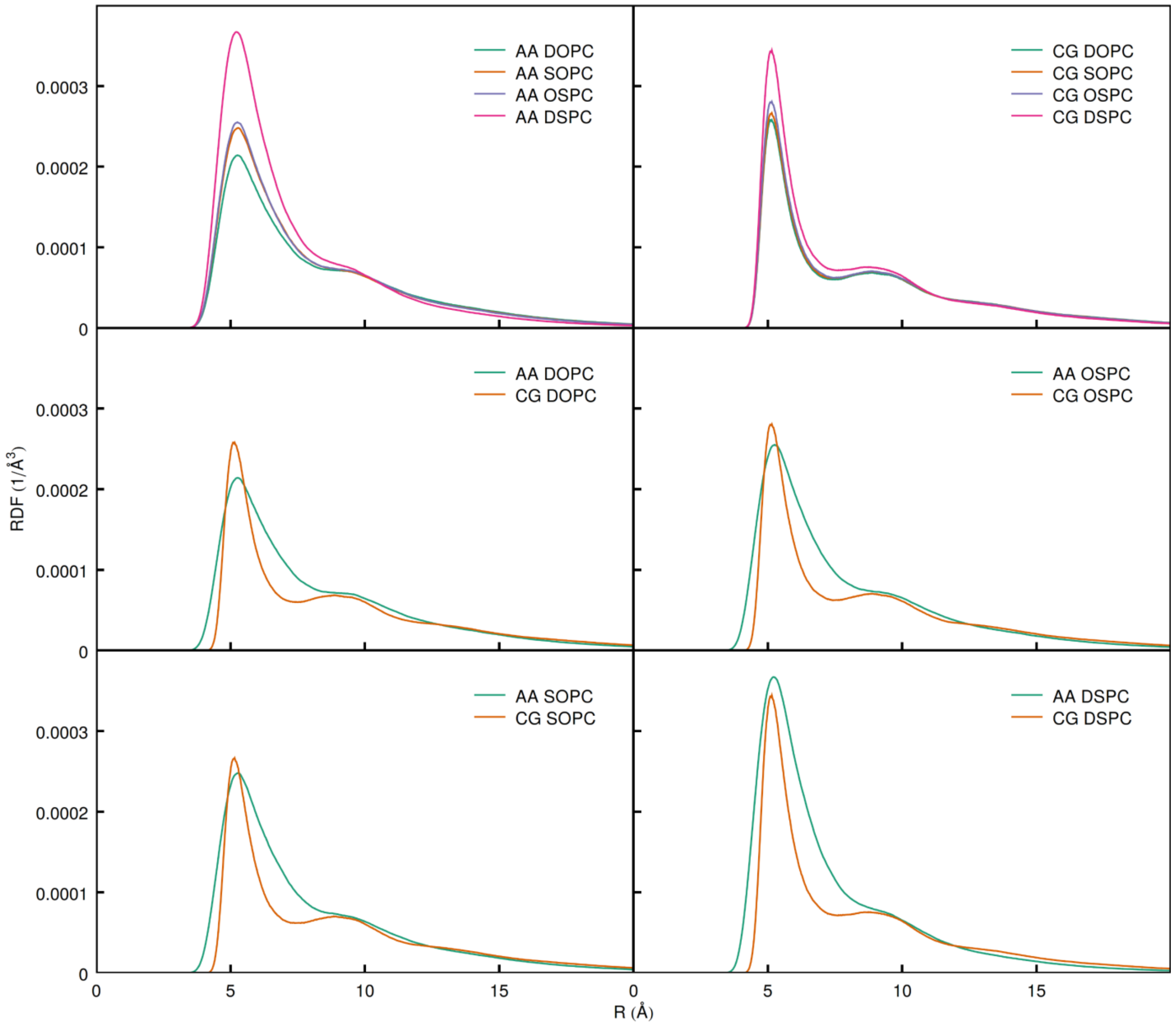


**Figure S11.** Radial distribution functions for chain-end beads C4A and C4B belonging to the same molecule. AA trajectory snapshots were coarse grained prior to the calculation.

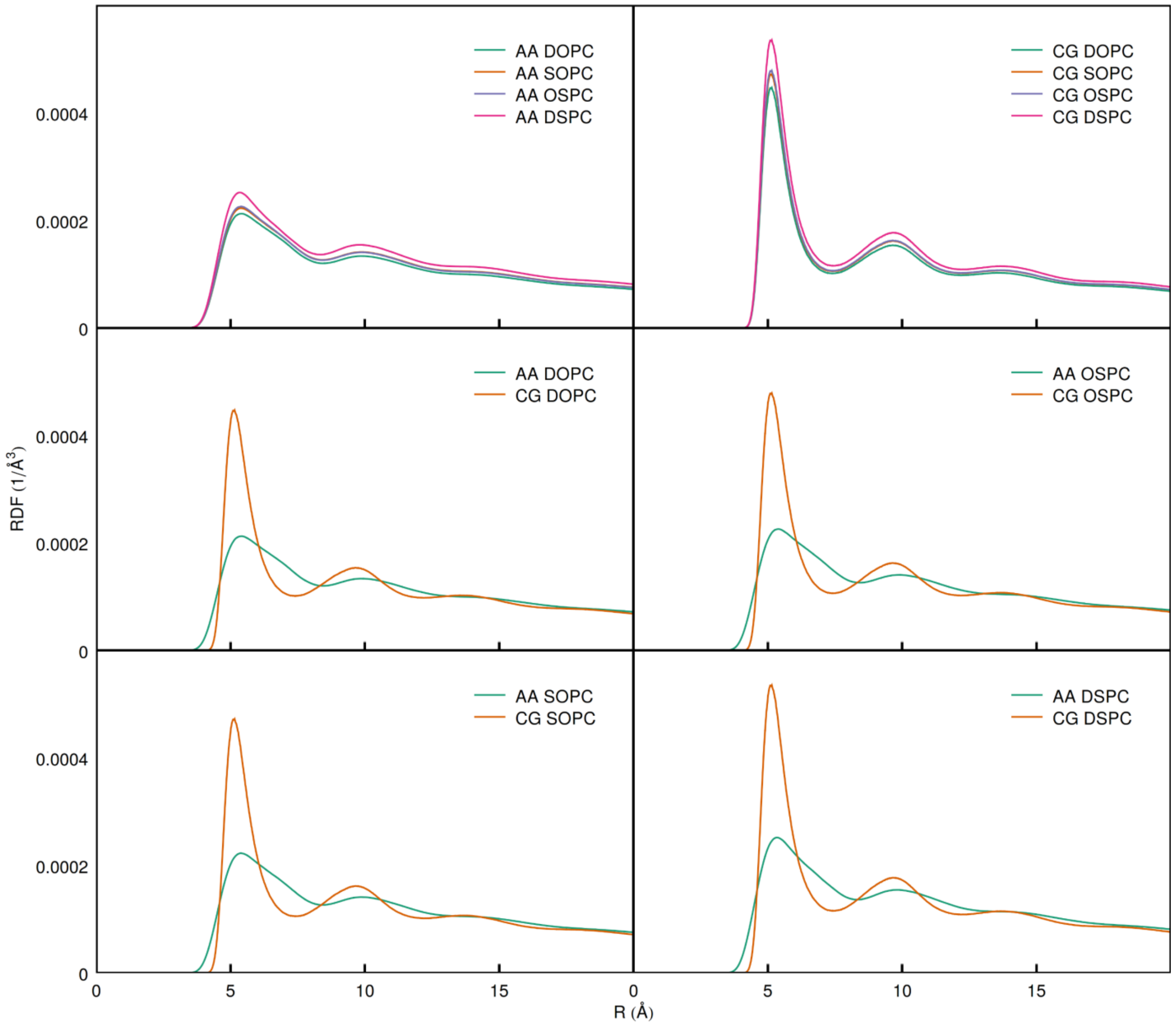


**Figure S12.** Radial distribution functions for chain-end beads C4A and C4B belonging to the same molecule as well as to the different molecules of the bilayers. AA trajectory snapshots were coarse grained prior to the calculation.

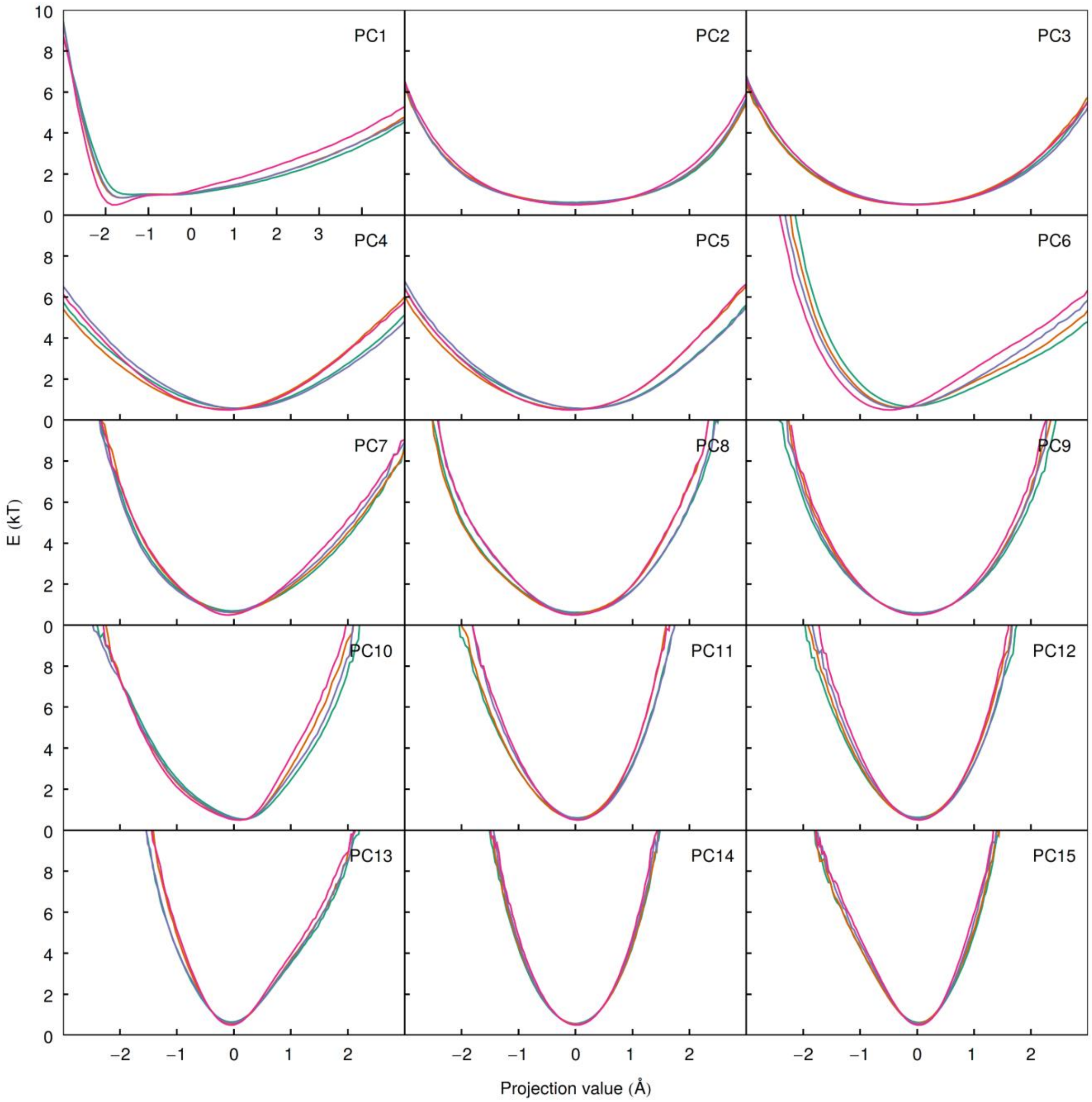


**Figure S13.** Effective potentials associated with the conformational changes along PC1–PC15 of the AA PC basis. Data for DOPC, SOPC, OSPC and DSPC are in marine, orange, violet and magenta, respectively.

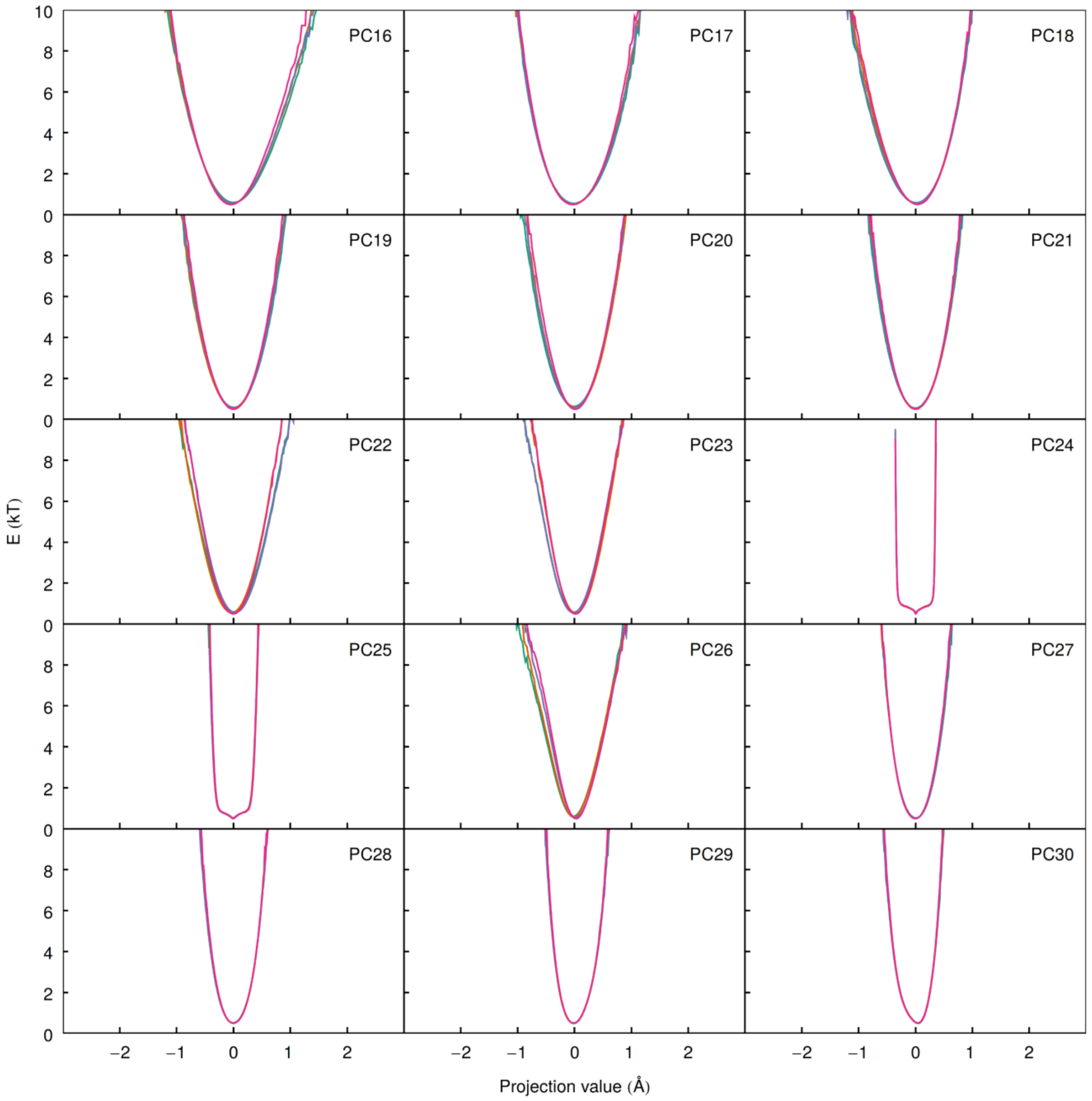


**Figure S14.** Effective potentials associated with the conformational changes along PC16–PC30 of the AA PC basis. Data for DOPC, SOPC, OSPC and DSPC are in marine, orange, violet and magenta, respectively. PC24 and PC25 correspond to relative motions of the choline moiety atoms and are consequently limited in amplitude.

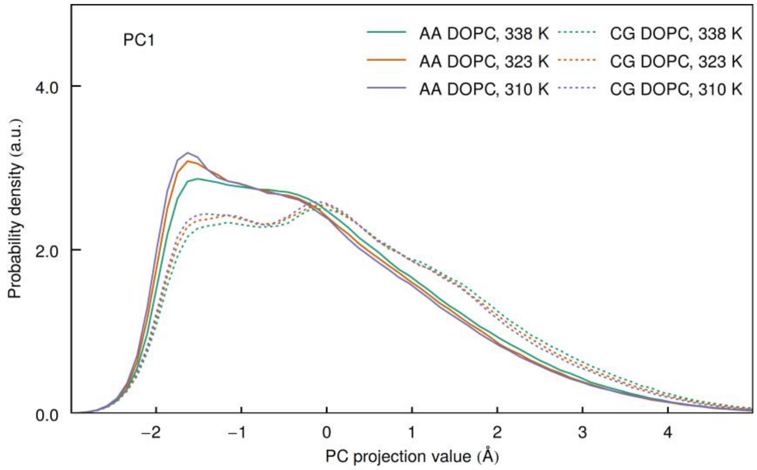


**Figure S15.** Influence of temperature on the distributions of the PC1 projections in the same PCA eigenvector basis.

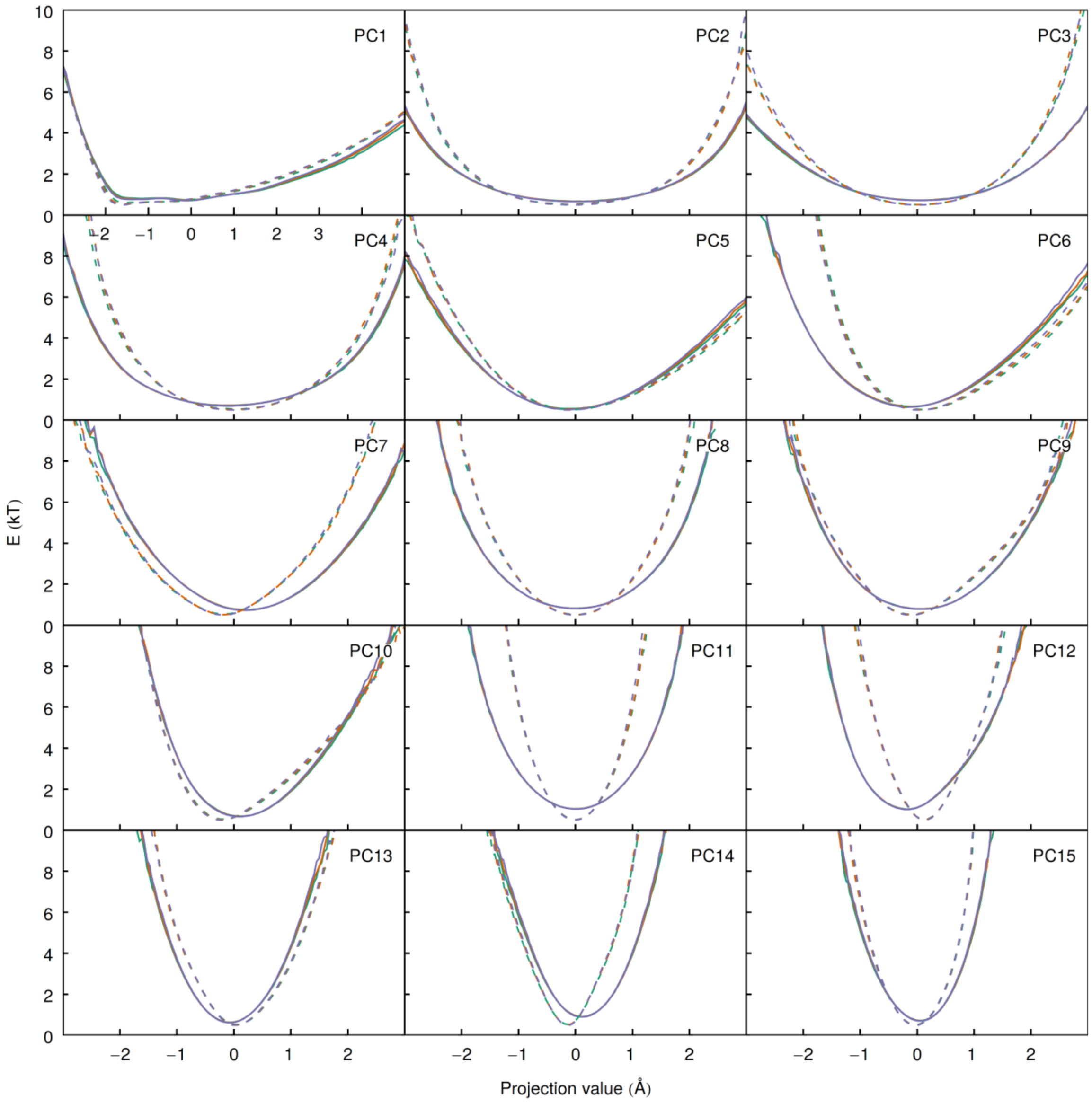


**Figure S16.** Effective potentials associated with the conformational changes along PC1–PC15. Data for 338 K, 323 K and 310 K are in marine, orange and violet, respectively. Data for AA and CG simulations are depicted with solid and dashed lines, respectively.

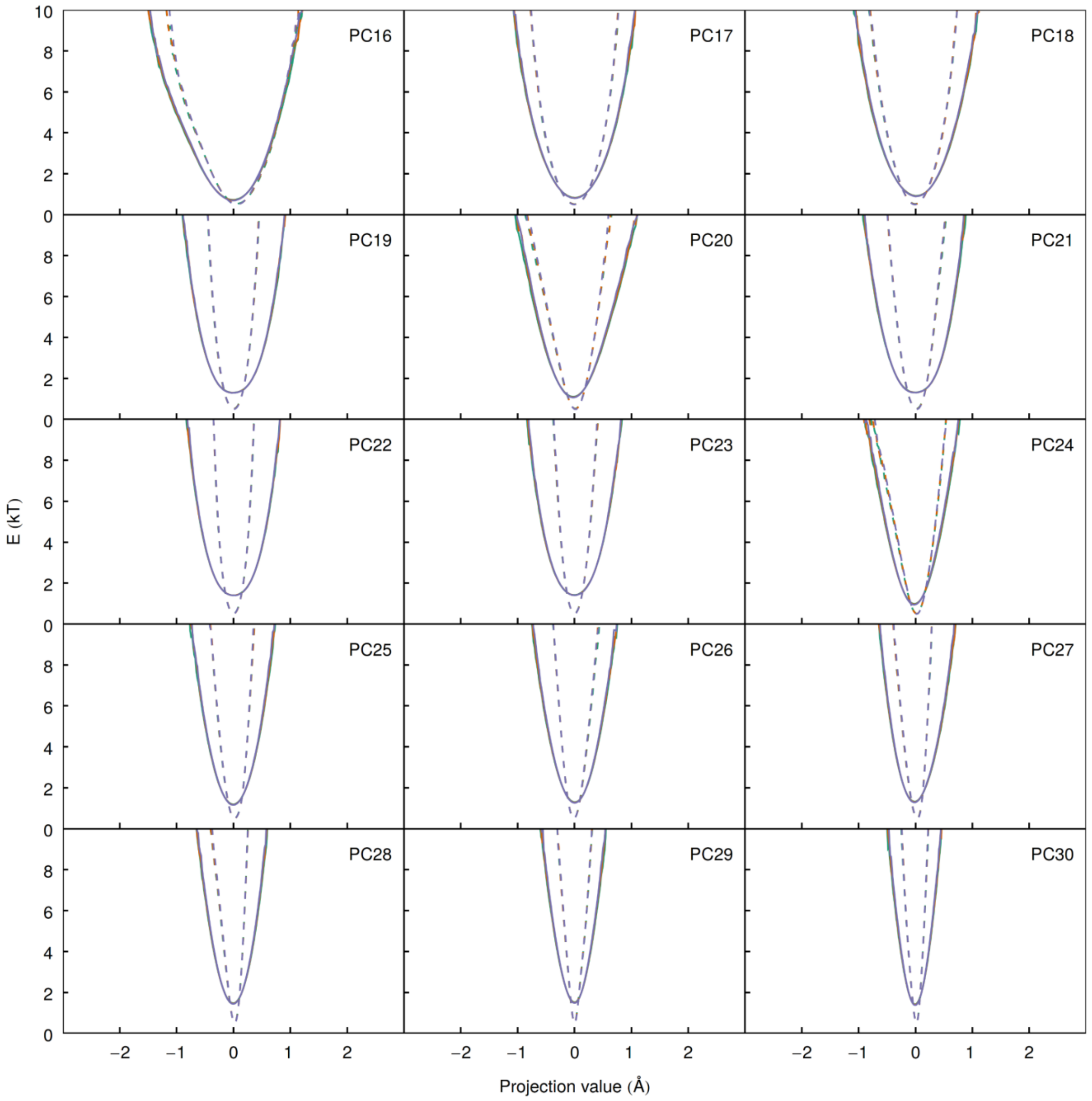


**Figure S17.** Effective potentials associated with the conformational changes along PC16–PC30. Data for 338 K, 323 K and 310 K are in marine, orange and violet, respectively. Data for AA and CG simulations are depicted with solid and dashed lines, respectively.

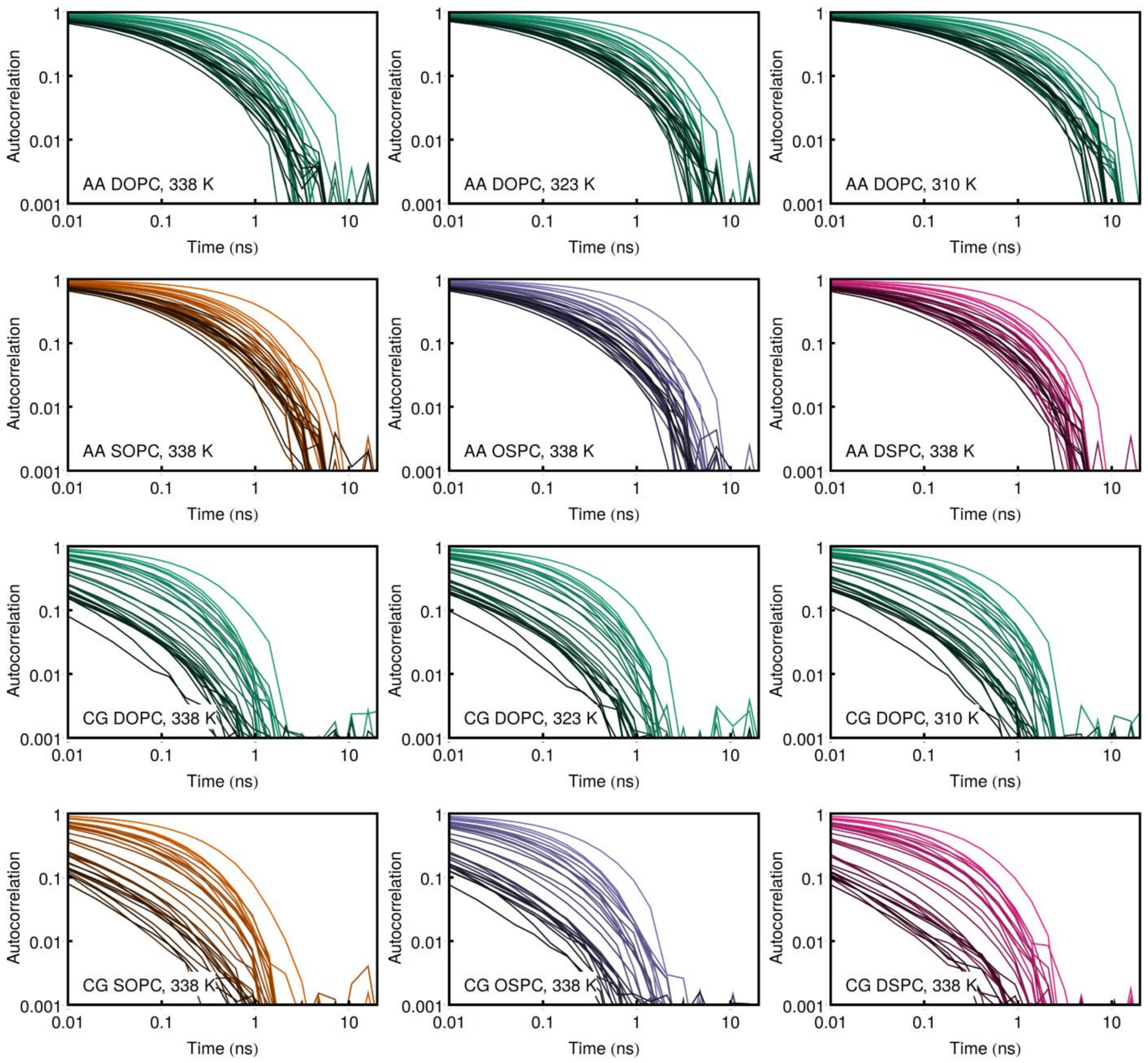


**Figure S18.** Autocorrelation of the projections on different principal components in common CG PC basis as a function of time lag. The color is gradually changed towards black for higher mode numbers.

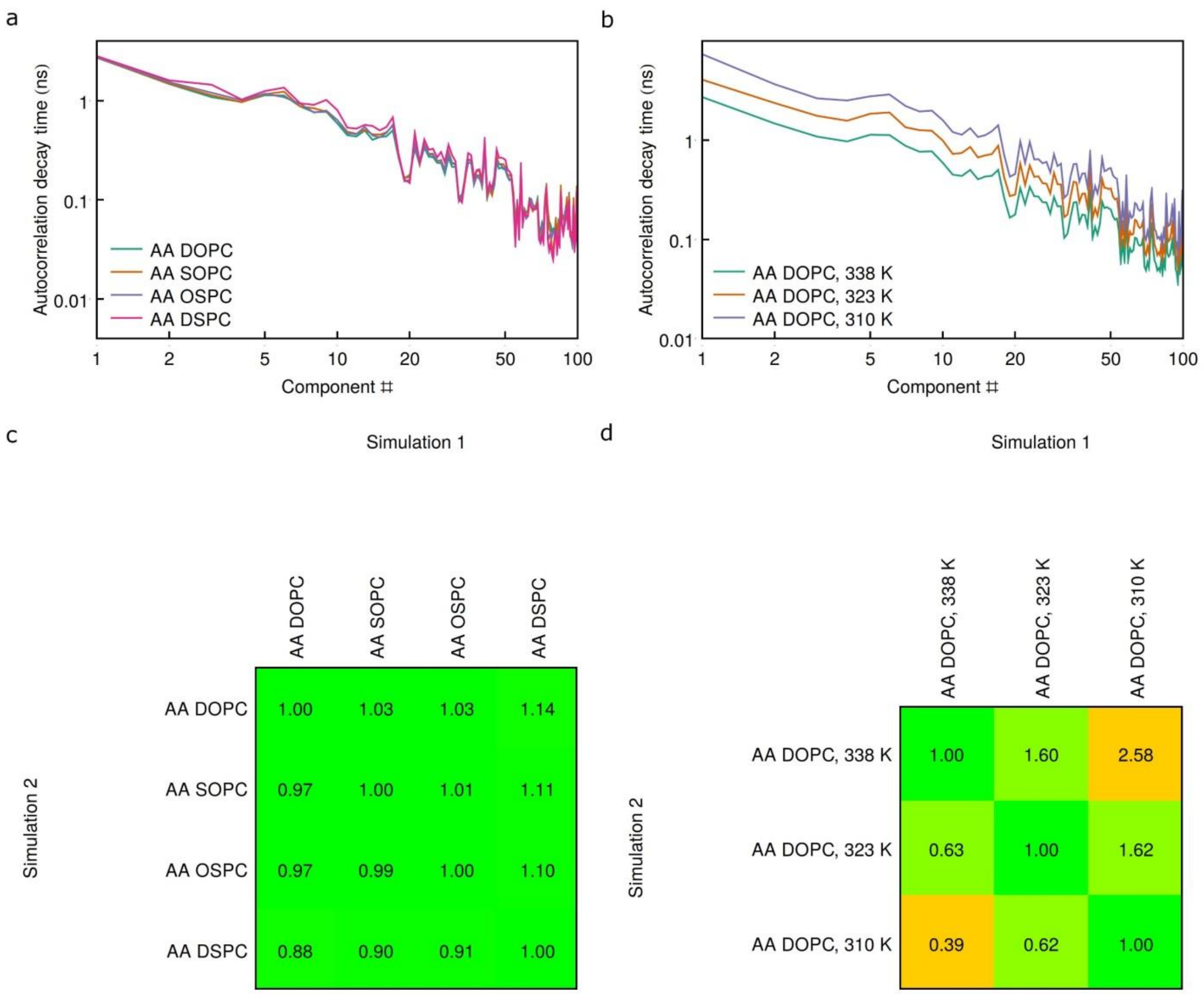


**Figure S19.** Characteristic autocorrelation decay times for different lipids (left) and for DOPC simulations conducted at different temperatures (right). The principal components analysis was conducted for the six trajectories in the joint AA PCA basis.
